# One-Pot Synthesis and Enhanced Vis-NIR Photocatalytic Activity of NiTiO_3_/TiO_2_ Templated by Waste Tobacco Stem-Silks

**DOI:** 10.3390/nano15020080

**Published:** 2025-01-07

**Authors:** Weidong Yuan, Xiaohong Chen, Yi Zhao, Ying Fang, Han Zhang, Daomei Chen, Jiaqiang Wang

**Affiliations:** School of Chemical Sciences & Technology, School of Materials and Energy, Yunnan Provincial Center of Technology Innovation for New Materials and Equipment in Water Pollution Control, Institute of International Rivers and Eco-Security, Yunnan Province Engineering Research Center of Photocatalytic Treatment of Industrial Wastewater, Yunnan University, Kunming 650091, China; yuanweidong@itc.ynu.edu.cn (W.Y.); chenxiaohong_qps6@stu.ynu.edu.cn (X.C.); 12022216197@stu.ynu.edu.cn (Y.Z.); fangying@stu.ynu.edu.cn (Y.F.); zhanghan2@stu.ynu.edu.cn (H.Z.); dmchen@ynu.edu.cn (D.C.)

**Keywords:** NiTiO_3_/TiO_2_, tobacco stem-silks biotemplate, Vis-NIR photocatalytic degradation of antibiotics, one-pot synthesis, Z-scheme heterojunction

## Abstract

Synthesis of the photocatalysts with near-infrared light response usually involves upconversion materials or plasmon-assisted noble metals. Herein, NiTiO_3_/TiO_2_ was synthesized by using waste tobacco stem-silks as biotemplates and tetra-tert-butyl orthotitanate and nickel nitrate as precursors in a one-pot procedure. NiTiO_3_(1.0)/TiO_2_(TSS) with a mass percent of Ni 1.0% exhibited very high visible-light photocatalytic efficiency in photodegradation of tetracycline hydrochloride (TC), which is 8.0 and 2.3 times higher than TiO_2_ prepared without templates and TiO_2_(TSS) prepared without Ni, respectively. Interestingly, NiTiO_3_(1.5)/TiO_2_(TSS) even exhibited good activity under NIR light (λ = 840~850 nm) without upconversion materials or plasmon-assisted noble metals, which is 2.8 and 2.2 times than TiO_2_ prepared without templates and TiO_2_(TSS), respectively. The boosting photocatalytic activity has been shown to be attributed to efficient charge separation and transfer across a direct Z-scheme heterojunction between NiTiO_3_ and TiO_2_ and enhanced light-harvesting ability of special flaky structure reduplicated from tobacco stem-silks. This reported strategy provides a new idea for the multifunctional utilization of waste tobacco stem-silks and the synthesis of novel photocatalysts for the potential application in wastewater treatment.

## 1. Introduction

Semiconductor-based photocatalysis has attracted significant attention in recent years due to its wide applications in solar energy conversion and environmental purification [1,2,3,4,5]. To efficiently utilize solar energy, it is necessary to develop novel photocatalytic systems that can operate effectively under visible light and even near-infrared (NIR) range irradiation [6,7,8]. Most photocatalysts could not serve as candidates for this application due to their relatively larger band gaps. To date, only very few photocatalytic systems, including the band gap engineering of composites, the use of doping semiconductors with upconversion materials or plasmon-assisted NIR responses, have been reported to possess photoactivity under NIR light [9,10,11]. For example, carbon dots modified with WO_2_-Na_x_WO_3_ composites [12], N-doped In_2_TiO_5_ [13], and carbon nanotube/CdS [14] are enhanced with NIR photocatalytic hydrogen evolution by band gap engineering. Upconversion photocatalysts such as Er^3+^-Yb^3+^ co-doped TiO_2_ [9], NaYF4:Yb, Tm@TiO_2_ [11] and BiErWO_6_ [15] have been reported as solar light efficient catalysts. However, the efficient utilization of NIR light is limited, owing to their very narrow absorption band of light at 980 nm [9,11], which results in low photocatalytic efficiency. Additionally, photocatalysts with plasmonic effects are often complex and involve using expensive noble metals [10]. Thus, it is highly desirable to develop novel vis-NIR photocatalysts for environmental purification [16].

NiTiO_3_ has been found to promote the optical absorption property of its composite because it is a sensitizing material with a relatively small band gap of about 2.14–2.50 eV [17]. Co-doped g-C_3_N_4_/NiTiO_3_ composites [18], Ag-loaded NiTiO_3_:V nanorods [19], NiTiO_3_/Ag_3_PO_4_ composites [20], and NiTiO_3_/Cd_0.5_Zn_0.5_S [21] have shown excellent photocatalytic activities under visible light irradiation. Furthermore, NiTiO_3_/g-C_3_N_4_ composites corresponded with the Z-scheme heterojunction, which can improve the photogenerated carrier migration efficiency [22]. The Z-scheme heterojunction has been reported in forms such as SrTiO_3_-FeS-CoWO_4_ [23], g-C_3_N_4_/TiO_2_ [24], and Cd_0.5_Zn_0.5_S/NiTiO_3_ [25]. In addition, BiO_2-x_ nanosheets doped by Ni^2+^ exhibit good photocatalytic activity under UV, visible, and near-infrared irradiation [7]. As far as we know, the photocatalytic activities of NiTiO_3_ or its composite under NIR light have not been explored. Thus, it can be a potentially good candidate for performing photochemical reactions under even NIR range irradiation.

In recent years, biotemplating has been developed as an effective strategy, with advantages such as the ability to obtain morphology-controllable materials with structural specificity and related unique functions, as well as biogenic-doped chemical elements [26,27]. Moreover, biotemplate materials are more economical and environmentally benign, making them incomparable with traditional chemical template methods, attracting more and more attention [28]. In our group, the skins of tomatoes, bulb onions, grapes, and garlic bulbs [29] were used to synthesize anatase mesoporous titania with novel morphologies, which only realized the replication of the morphologies. Moreover, in mesoporous TiO_2_/SiO_2_ composite templated by aquatic plant leaves [30], ZnIn_2_S_4_ photocatalyst derived from hydrilla [31] and graphene-wrapped ZnO nanospheres from cyanobacterial cells [32] exhibited varied UV- and visible-light photocatalytic activities. Especially, biotemplate materials are rich sources of various non-metallic elements (C, N, S, P, etc.), such as mesoporous N-S-C tri-doped TiO_2_ photocatalyst templated by expired egg white [33], which can be easily integrated into non-metallic doped photocatalysts without adding chemical precursors. In addition, TiO_2_ and graphitic carbon composites with hierarchical pore structures were synthesized using Staphylococcus aureus (ATCC6538) as the biotemplate to modify Degussa P25 TiO_2_ powder, realizing carbon self-doping and the enhancement of visible-light photocatalytic activity [34]. However, metal ion doping is an effective method to improve the catalytic activity of TiO_2_. Metal ions are more active and easier to excite electrons, which can change the energy level structure of TiO_2_ and expand its light response range. For example, three-dimensional TiO_2_ hollow spheres doped with rare earth elements were successfully synthesized via a nonhydrolytic sol–gel method using lotus pollen as a biotemplate and a cationic surfactant as a contemplate, realizing additional metal ion doping while replicating the morphology of biotemplate [35]. In addition, bio-inspired nanoarchitectured hybrid systems towards enhanced far red-to-near-infrared solar photocatalysis have been explored in recent years. A typical photocatalytic unit with butterfly wings’ 3D micro/nanoarchitectures demonstrates obvious far red-to-NIR photocatalysis enhancement [36]. Therefore, bio-inspired methods could be potential for the synthesis of photocatalysts with visible light and even NIR light response [37].

Tetracycline antibiotics (TCS) are the most common antibiotics in the world, and TC accounts for the largest proportion of TCS. TC has the advantages of low price, good quality, and high synthetic purity. However, TC is difficult to metabolize and absorb by animals and can remain in water for a long time. The uncontrolled use and arbitrary release of TC into the environment pose a certain threat to the ecological environment. TC is a relatively stable and difficult to degrade organic substance. At present, there are various technologies for removing TC from water, including adsorption [38], microbial degradation [39], electrolysis [40], and photocatalytic degradation [41]. Among them, photocatalytic degradation generally with high degradation efficiency is considered a green and sustainable technology for the removal of TC. On the other hand, a mass of biomass material of tobacco stem is disposed of worldwide annually and causes a waste of natural plant resources and serious environmental pollution.

Obviously, it is a relatively developed method to enhance the photocatalytic performance of TiO_2_ using biotemplates and transition metal doping, but there are a few aspects to explore regarding the effect of the simultaneous use of biotemplates and metal ion doping on its photocatalytic degradation efficiency. Herein, NiTiO_3_/TiO_2_ was successfully synthesized by using waste tobacco stem-silks as biotemplates via the facile one-pot method. It was found that the novel hybrid-nanostructured NiTiO_3_/TiO_2_ heterojunction was formed instead of conventional Ni-doped TiO_2_ or NiO/TiO_2_. The photocatalytic activities of all prepared photocatalysts were evaluated by visible and NIR light-driven photocatalytic degradation of TC. The strategy we reported provides a new idea for the multifunctional utilization of waste tobacco stem-silks and the synthesis of novel vis-NIR photocatalysts for the potential application in tetracycline treatment in wastewater.

## 2. Materials and Methods

### 2.1. Chemicals and Materials

The tobacco stem-silks as bio-template were obtained from Kunming Cigarette Factory. Tetra-tert-butyl orthotitanate (TBOT) and nickel nitrate hexahydrate (Ni(NO_3_)_2_·6H_2_O) were used as sources of titanium and nickel. They were both procured from Adamas-beta in Shanghai. Ethanol and glutaraldehyde were purchased from Xilong Science in Chengdu. All chemicals were of analytical grade and were used as received.

### 2.2. Treatment of Tobacco Stem-Silks

Usually, it is necessary to pretreat bio-template materials. In the synthetic procedure, the tobacco stem-silks were treated with glutaraldehyde solution (5%) for 24 h. After washing with distilled water, the stem-skills were soaked in HCl (5%) for 24 h, followed by ethanol dehydration and drying at 90 °C for 12 h.

### 2.3. Synthesis of NiTiO_3_/TiO_2_ (TSS)

**Scheme 1:**
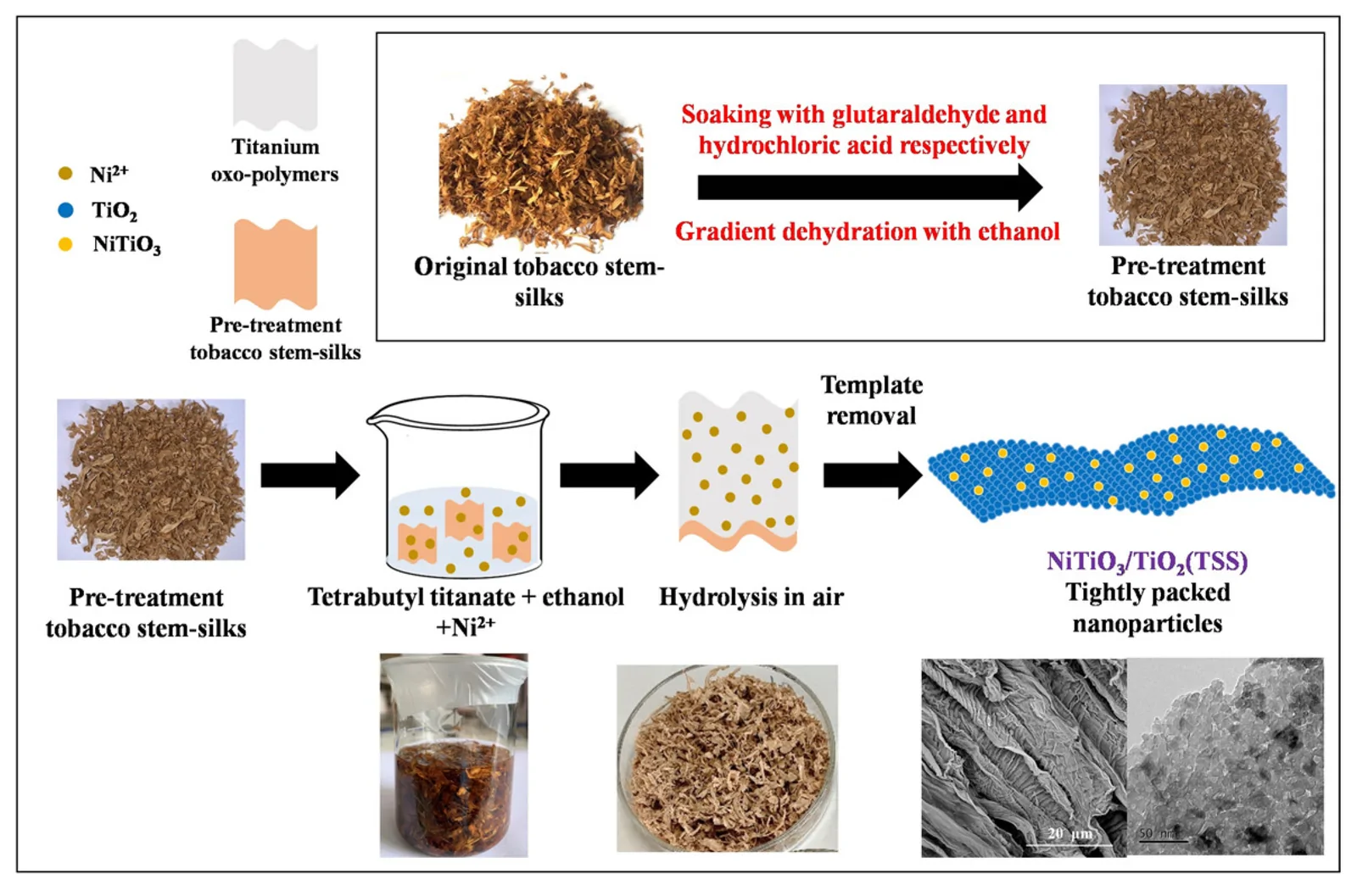
Schematic illustration of NiTiO_3_/TiO_2_(TSS) synthesis via one-pot.

Biotemplating method has been applied to the synthesis of a series of NiTiO_3_/TiO_2_(TSS) photocatalysts in this work. The synthesis of the photocatalysts via a replication route, using two-dimensional tobacco stem-silks with flaky structures as templates and TBOT and Ni(NO_3_)_2_·6H_2_O as source materials. The synthesis procedure of NiTiO_3_/TiO_2_(TSS) is shown in Scheme 1. The pretreated tobacco stem-silks (2 g for every sample) was immersed in precursor solutions of Ni(NO_3_)_2_·6H_2_O, TBOT (5 mL) and ethanol (50 mL) for 24 h. After the precursor solution was poured out, the tobacco stem-silks were directly exposed to air for 24 h. The hydrolyzed tobacco stem-silks were subsequently calcined (2 °C/min) at 450 °C for 10 h. According to the different mass percent of Ni/Ti, the samples were denoted NiTiO_3_(X)/TiO_2_(TSS). TSS meant tobacco stem-silks TiO_2_ and X meant the mass percent of Ni/Ti. For the purpose of a contrast analysis, four other samples were prepared: (1) TiO_2_(TSS) was prepared with the same Ti content as NiTiO_3_(X)/TiO_2_(TSS); (2) TiO_2_ was prepared without templates and Ni added; (3) NiTiO_3_ was prepared by a reported method with processes described in Ref. [42]; (4) Ni/TiO_2_ was prepared without templates. The mixed solution of Ni(NO_3_)_2_·6H_2_O, TBOT (2 mL), and ethanol (20 mL) was vigorously stirred for 2 h and then exposed to air for 24 h. The hydrolyzed product was subsequently calcined (2 °C/min) at 450 °C for 10 h.

### 2.4. Characterization of the Prepared Photocatalysts

To characterize the prepared samples, a variety of surface and analytical techniques were employed. The crystalline phase was examined using the X-ray powder diffraction (XRD) method in the 2θ range of 20–70°. This analysis was conducted with a Rigaku TTRAX III operating at 72 W with Cu Kα radiation. Morphological and structural details were determined by the field-emission scanning electron microscopy (FE-SEM, FEI Quanta 200 US) and transmission electron microscopy (TEM, Hitachi H-800 Japan). Additionally, Brunauer–Emmett–Teller (BET) surface areas were measured using a Micromeritics Tristar II 3020 Surface Area and Porosity Analyzer. Chemical composition analysis was facilitated by X-ray photoelectron spectroscopy (XPS, Thermo Fisher Scientific K-Alpha+ US) with mono Al Kα radiation (hv = 1486.6 eV). High-resolution XPS scans (HR-XPS US) were performed at PE = 30 eV with a step size of 0.1 eV. Furthermore, UV-Vis diffuse reflectance spectra and solution absorbance were characterized on a Shimadzu UV-2600 photometer. Photocurrent responses (CHI 760E) and electrochemical impedance spectra (EIS, Metrohm PGSTAT 302N Switzerland) were measured in a standard three-electrode system, with the prepared sample electrode serving as the working electrode, Pt wire as the counter electrode, and a saturated Ag/AgCl electrode as the reference electrode. The electrolyte used was a 0.5 M Na_2_SO_4_ aqueous solution, and a 350 W xenon lamp was employed as the light source. For further analysis, photoluminescence (PL) spectroscopy was conducted using a HitachiF-7000 Japan fluorescence spectrometer. Finally, electron paramagnetic resonance (EPR) spectra were collected at room temperature (Frequency, 9.85 GHz; Power, 20 mW; Modulation A Frequency, 100 kHz) with a Bruker A300- Germany spectrometer.

### 2.5. Photocatalytic Activity

The photocatalytic activity of TiO_2_, TiO_2_(TSS) and NiTiO_3_/TiO_2_(TSS) samples were evaluated by the degradation of TC (10.0 mg/L) in aqueous solution under visible light. The experiments were performed in a photochemical reactor system. 30 mg of photocatalyst was dispersed in 50 mL of TC solution in a glass vessel (an internal volume of 50 mL) with a flat bottom made from quartz. The mixed solution was under vigorous stirring in the dark for 60 min to reach an equilibrium adsorption state. Afterwards, the reactor was irradiated with visible light (LED lamp: 5 W of visible light power, light from bottom to top, and wavelength ranged from 420 nm to 800 nm) under continued stirring. At time intervals of 10 min from 0 to 50 min, 1 mL of the reaction solution was collected and the suspended photocatalyst was filtered out by a 0.45 μm membrane filter of aqueous phase. Then, TC in the collected solution was determined by high performance liquid chromatography (HPLC, Agilent 1260 Infinity) using an Agilent C18 chromatographic column (4.6 × 150 mm, 5 μm). Mobile phase A: aqueous solution of oxalic acid (0.01 mol/L). Mobile phase B: mixed solution of methanol and acetonitrile (*V*/*V* = 1/3). The gradient solvent was as follows: Table S1. The injunction volume was 10 μL. The flow rate was set at 1.000 mL/min, and the column temperature was maintained at 25 °C. The signal of UV detector was set at the wavelength of 350 nm and 357 nm. Equation (1) was used to calculate the removal rate of TC, where C_0_ is initial absorption of TC at 357 nm and C is instantaneous absorption of TC at 357 nm. Equation (2) was used to calculate the first-order rate constant k (min^−1^), where Ce is the equilibrium adsorption of TC at 357 nm, t is the irradiation time, and C is the instantaneous adsorption at time t. The standard curve for TC quantification by HPLC is showed in Figure S1. The R2 of the standard curve is 0.99988, which can be used for quantitative calculation of TC.

Similarly, 15 mg of photocatalyst was dispersed in 20 mL of TC solution (10.0 mg/L) in a glass vessel (an internal volume of 20 mL) with a flat bottom made from quartz. The mixed solution was under vigorous stirring in the dark for 150 min to reach an equilibrium adsorption state. Afterwards, the reactor was irradiated with NIR light (LED lamp: 5 W of NIR light power, light from bottom to top, and wavelength ranged from 840 nm to 850 nm) under continued stirring. At time intervals of 60 min from 0 to 240 min, 1 mL of the reaction solution was collected and suspended photocatalyst was filtered out by a 0.45 μm membrane filter of aqueous phase. Then, TC in the collected solution was determined by HPLC.

Removal yield (%) = (C_0_ − C)/C_0_ 100% (1)

ln (Ce/C) = kt(2)

The intermediates and transformation products of the photocatalytic oxidation of TC were analyzed by an ultra-high performance liquid chromatography-tandem mass spectrometry (UPLC–MS/MS) method. The instrument used was UPLC–MS/MS, Waters Acquity UPLC-Xevo TQ, USA). A BEH C18 column (2.1 mm × 50 mm, 1.7 μm) was the chromatographic separation column at 30 °C. The mobile phase was a mixture of 10.0% acetonitrile and 90.0% formic acid (0.1%) at a flow rate of 0.3 mL/min. The scanning mass range was from 200 to 500 *m*/*z* using ESI in the positive-ion mode.

## 3. Results and Discussion

### 3.1. Synthesis and Photocatalysts Characterizations

Different from traditional solvothermal route, the synthesis of NiTiO_3_/TiO_2_(TSS) via a facile and simple one-pot method, using two-dimensional tobacco stem-silks with flaky structure as hard templates. As shown in Scheme 1, the pretreated tobacco stem-silks were directly immersed in the precursor solution of Ni and Ti sources at room temperature. The further sol–gel was carried out open to ambient air to allow very gradual diffusion of water into the system, which resulted in noticeable hydrolysis and formation of TiO_2_. Subsequently calcining achieved the removal of templates, the microstructure replication of tobacco stem-silks, and the formation of NiTiO_3_/TiO_2_. The synthetized TiO_2_(TSS) and NiTiO_3_/TiO_2_(TSS) photocatalysts were analyzed by the following characterizations.

**Figure 1:**
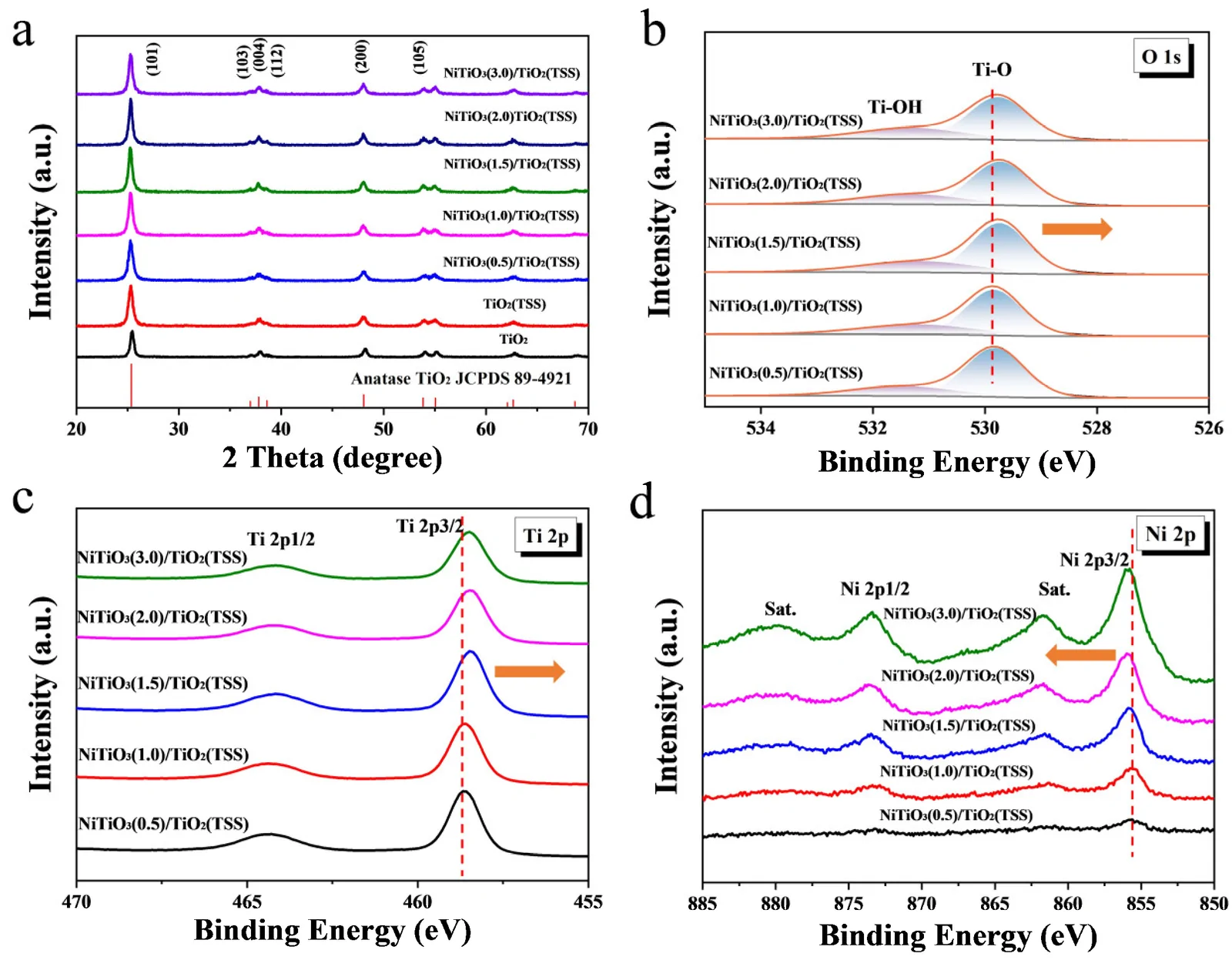
XRD patterns of TiO_2_ prepared without templates, TiO_2_(TSS), and NiTiO_3_/TiO_2_(TSS) (**a**). XPS spectra of O 1s, (**b**) Ti 2p, and (**c**) Ni 2p of (**d**) NiTiO_3_/TiO_2_(TSS).

The crystal structure of the prepared photocatalysts was analyzed and evaluated by XRD measurement in Figure 1a. The obvious diffraction peaks at 25.3°, 37.8°, 48.0°, 53.9°, 55.1°, 62.7° correspond to anatase TiO_2_ (101), (103), (004), (112), (200), (105), (211), (213), (204) crystal plane, respectively, indicating that TiO_2_ in all synthesized photocatalysts was the anatase phase (in accord with JCPDS 89-4921) without any other polymorphs. Similarly to those transition metal doping, the diffraction peaks of NiTiO_3_ were not observed, which was probably attributed to the uniform distribution or the low content of NiTiO_3_ [43]. To confirm the formation of NiTiO_3_, NiTiO_3_(5.0)/TiO_2_(TSS) was synthesized with the same synthetic procedure. As shown in Figure S2a, the diffraction peaks of NiTiO_3_ were observed in NiTiO_3_(5.0)/TiO_2_(TSS) sample, which verify the existence of NiTiO_3_. This is different from conventional reported biotemplate method in which Ni doping was usually conducted after the synthesis and only Ni doped TiO_2_ or NiO/TiO_2_ was formed.

**Figure 2:**
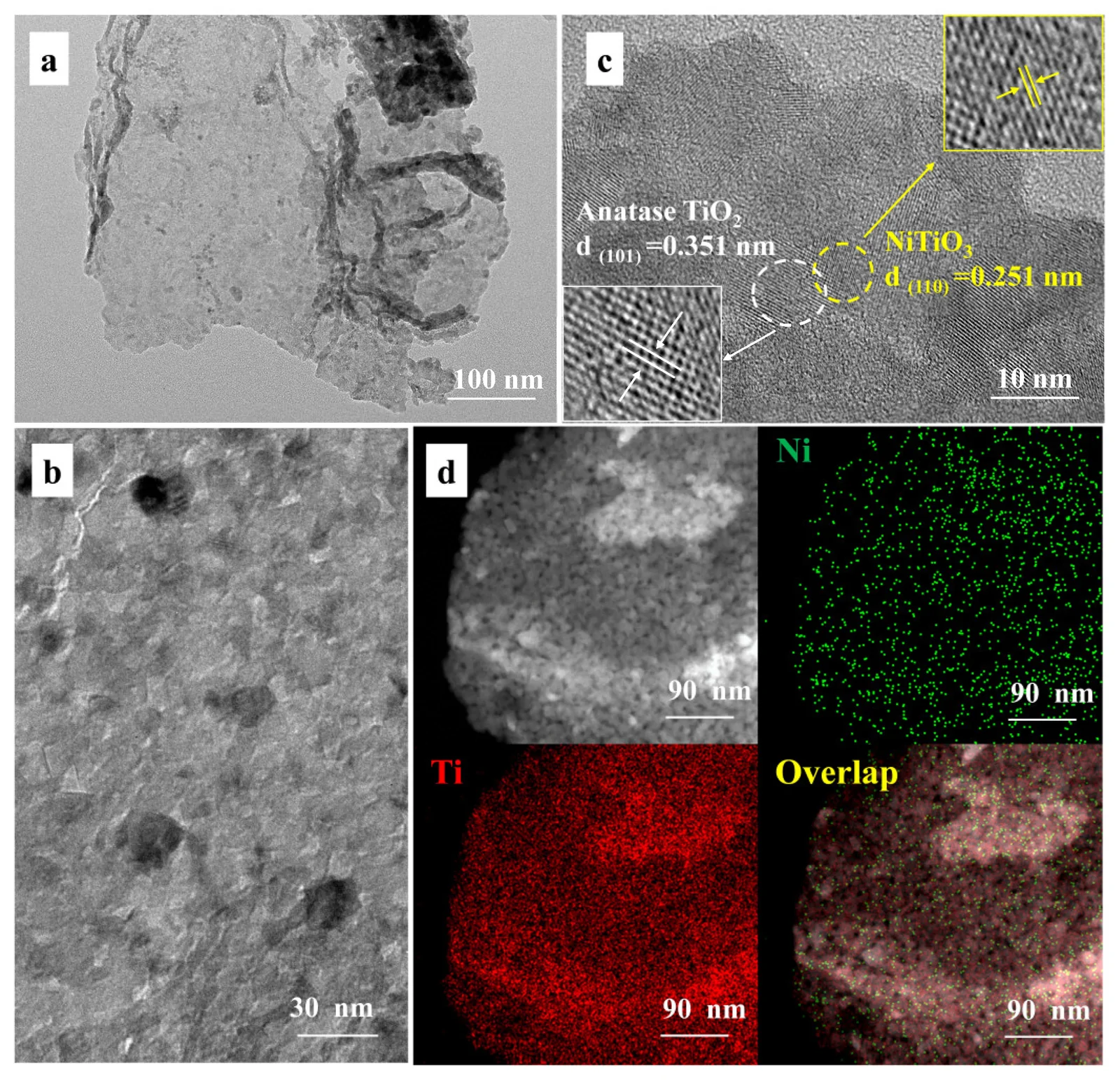
TEM images (**a**,**b**), HR-TEM image (**c**), and EDS element mapping of (**d**) NiTiO_3_(1.0)/TiO_2_.

To further study and understand the elemental composition and the chemical state of Ti and Ni in the synthesized NiTiO_3_/TiO_2_(TSS) photocatalysts, XPS analysis of NiTiO_3_/TiO_2_(TSS) was carried out. The survey spectra demonstrating the presence of O and Ti components was shown in Figure S3a. The XPS signal of the elemental Ni was not explicit in Figure 2b probably on account of the low content of NiTiO_3_ (X < 1.5). However, with the increasing content of Ni added, the signal of the elemental Ni was gradually explicit (X ≥ 1.5). Three carbon species were observed in the C 1s spectrum in Figure S3b. The species with binding energy of 284.8 eV and 286.4 eV could be attributed of carbon backbone (aromatic and aliphatic) and C-O bonds within the adventitious carbon contaminants which are commonly reported in samples that have been exposed to the atmosphere [44]. The specie with binding energy of 288.5 eV could be characteristic for O-C=O due to the residual oxidized organic species of biotemplate [45,46], which is probably related to the carbon species remaining after removal the tobacco stem-silks via calcination [47]. Figure 1b shows O 1s spectra decomposed of two subpeaks at 529.8 eV and 530.8 eV, corresponding to the Ti−O bonds in the lattice oxygen and the hydroxyl Ti-OH groups on the surface, respectively [48]. Figure 1c shows the Ti 2p spectra of NiTiO_3_/TiO_2_(TSS). Two characteristic peaks in the XPS spectrum of Ti 2p correspond to Ti 2p1/2and Ti 2p3/2 at binding energies of 458.6 eV and 464.2 eV, respectively [48,49], which can be attribute to the Ti^4+^ species, indicating that Ni atom is not doped into the TiO_2_ lattice in the form of substituting Ti^4+^. As displayed in Figure 1d and Figure S2b, the characteristic peaks of Ni 2p1/2 and Ni 2p3/2 were observed, along with two satellite peaks. The main peak at binding energy of about 855.9 eV for NiTiO_3_(X)/TiO_2_(TSS), indicating the similar oxidation state of Ni in the prepared catalysts. It should be noted that this result can exclude the formation of NiO, with a banding energy at about 854.0 eV [42]. And NiO exhibits the typical multiplet-split peaks, which were not observed in all synthesized NiTiO_3_/TiO_2_(TSS) [48,50]. As a result, different from formed oxides of traditional Ni doping, NiTiO_3_ was formed by the biotemplate strategy we propose, which is unexpected and can be attributed to the unique structure of waste tobacco stem-silks and the slow hydrolysis exposed to the air in the synthesis process. In addition, it is worthwhile to note that the binding energies of both Ti 2p and O 1s show a negative shift with the increasing content of NiTiO_3_, whereas the binding energies of Ni 2p show a positive shift. The binding energies with negative/positive shifts indicate that TiO_2_ and NiTiO_3_ have strong electronic interactions [48]. It has been proven that the strong interactions between TiO_2_ and NiTiO_3_ can widen the light absorption range and help to improve photocatalytic activities of the catalysts [51], which will be discussed later. Similar heterostructure systems have also been reported, such as NiTiO_3_/Ag_3_VO_4_ [17] and g-C_3_N_4_/NiTiO_3_ [18]. According to the above XPS results, the existence of NiTiO_3_ in NiTiO_3_/TiO_2_(TSS) is confirmed, while both the formation of NiO and the substitution of Ni^2+^ for Ti^4+^ in the TiO_6_ octahedrons can be excluded [43].

The microscopic morphology of TiO_2_ prepared without templates, TiO_2_(TSS), the original tobacco stem-silks, and NiTiO_3_(1.0)/TiO_2_(TSS) was characterized by SEM. It can be seen from the images in Figure S4a that TiO_2_ prepared without templates was tightly piled up by TiO_2_ particles, with a spherical microstructure and an irregular order. The waste tobacco stem-silks were used as biotemplates in the synthesis of TiO_2_(TSS) and NiTiO_3_(1.0)/TiO_2_(TSS). It can be seen from Figure S4b–d that the flaky structure of tobacco stem-silks was preserved, which was completely different from TiO_2_ prepared without templates, indicating the successful replication of micro-architectures of tobacco stem-silks. According to the results, the developed templating method provides a new and reliable approach to synthesizing photocatalysts with special and complex architectures using natural materials. Moreover, the EDX image (Figure S5) of NiTiO_3_(1.0)/TiO_2_(TSS) verified the coexistence of C, O, Ni, and Ti elements.

**Figure 3:**
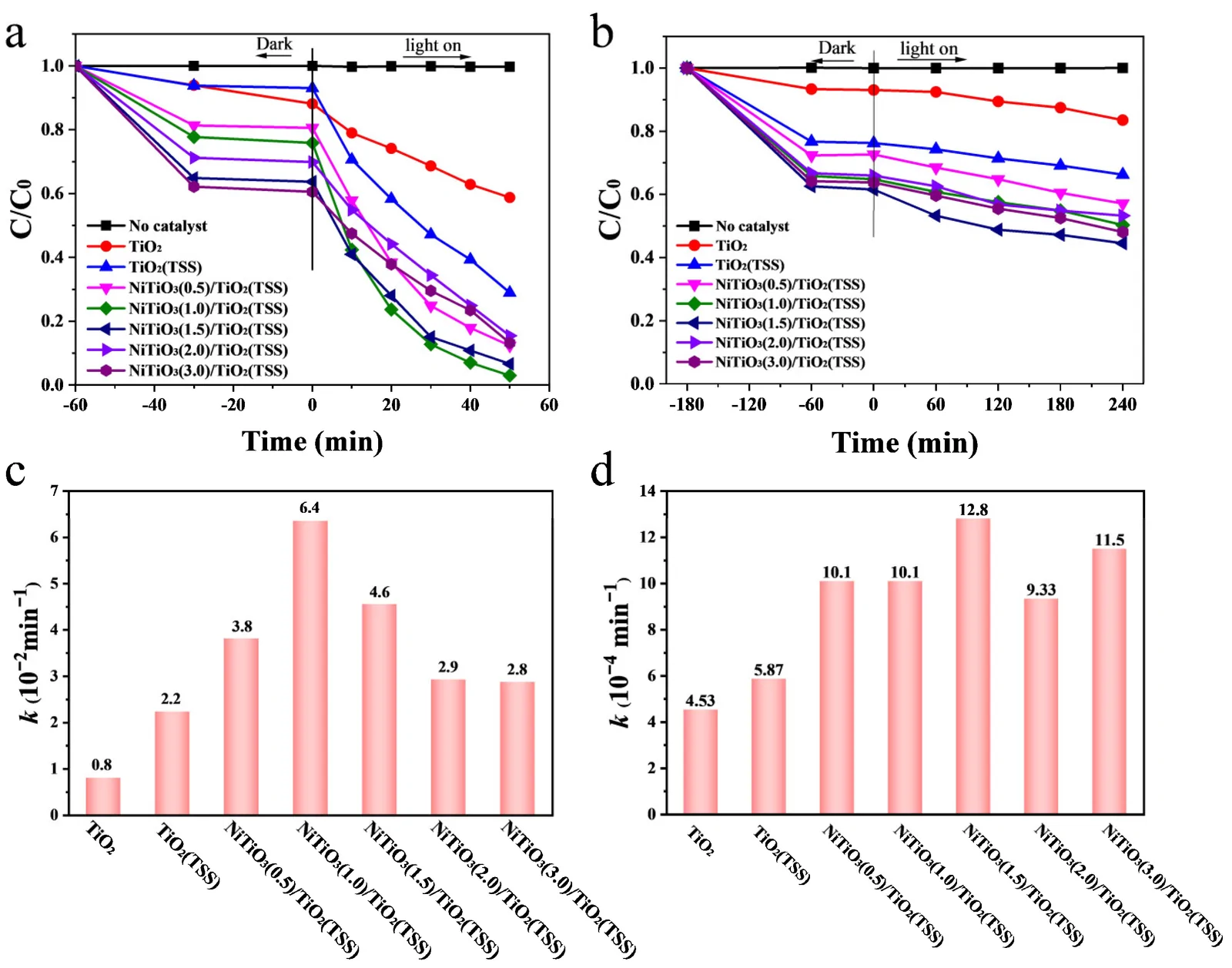
Removal curves of TC over the prepared photocatalysts under visible-light irradiation (**a**) and under NIR (840–850 nm) light irradiation (**b**). Apparent first order rate constant k (min^−1^) for TC photocatalytic degradation over the prepared photocatalysts under visible-light irradiation (**c**) and NIR (840–850 nm) light irradiation (**d**).

The microstructure of TiO_2_, TiO_2_(TSS), and NiTiO_3_(1.0)/TiO_2_(TSS) was further analyzed via TEM and HR-TEM. It was found that the micro-architectures of tobacco stem-silks was successfully replicated and the majority of organic components of tobacco stem-silks was removed, which can be seen clearly in Figure 3a,b and Figure S6a,c. Figure 2c shows the HR-TEM image of NiTiO_3_/TiO_2_ heterostructure and clearly shows the lattice fringes corresponding to the (101) (d101 0.352 nm) and the (110) (d110¼ 0.251 nm) crystallographic planes of TiO_2_ and NiTiO_3_, respectively, which further verifies the existence of NiTiO_3_. Energy-dispersive X-ray spectroscopy (EDS) mapping in Figure 2d for NiTiO_3_(1.0)/TiO_2_(TSS) showed a homogeneous distribution of metal elements within the photocatalyst, suggesting that the elements of Ni and Ti successfully diffuse deep inside the pore of tobacco stem-silks. Moreover, the HR-TEM images of TiO_2_ and TiO_2_(TSS) in Figure S6b,d verified the formation of TiO_2_, which was consistent with the results of XRD.

Finally, as shown in Figure S7, the nitrogen-gas adsorption–desorption analysis of as-prepared TiO_2_(TSS) and NiTiO_3_/TiO_2_(TSS) photocatalysts was conducted to study their textural properties and porosity distributions, which were in accordance with irreversible type IV adsorption isotherm (IUPAC classification) and H3 hysteresis loop. The results reveal that the structures of all photocatalysts are mesoporous solid and nonrigid aggregates of flaky particles. It can be seen from the pore size distribution in Figure S7 (insert) that there is a strong accumulation peak at 0–10 nm, indicating that there are mesoporous channels inside the photocatalysts. Some physicochemical properties of as-prepared photocatalyst are shown in Table S2.

### 3.2. Photocatalytical Degradation of Tetracycline

The visible-light photocatalytic activities of TiO_2_ prepared without templates, TiO_2_(TSS) and NiTiO_3_/TiO_2_(TSS) were evaluated by degradation of TC. To achieve real photocatalytic degradation, the adsorption yield of TC was assessed in the presence of photocatalysts. It was approximately 60 min that adsorption–desorption equilibrium was reached. As shown in Figure 3a, the efficiencies of TC removal over NiTiO_3_(0.5)/TiO_2_(TSS), NiTiO_3_(1.0)/TiO_2_(TSS), NiTiO_3_(1.5)/TiO_2_(TSS), NiTiO_3_(2.0)/TiO_2_(TSS), and NiTiO_3_(3.0)/TiO_2_ (TSS) were 87.7, 97.0, 93.4, 84.6, and 86.8%, respectively. Their removal efficiencies were all higher than those of TiO_2_ prepared without templates (41.2%) and TiO_2_(TSS) (71.1%).

Additionally, as shown in Figure S9a, the removal efficiency of NiTiO_3_(1.0)/TiO_2_(TSS) were higher than bare NiTiO_3_ (31.8%) and Ni/TiO_2_ (71.5%). To further evaluate the visible-light photocatalytic activities of the prepared catalysts, an apparent first order rate constant k (min^−1^) for photocatalytic degradation of TC was calculated and plotted in Figure S8a. It can be seen from Figure 3b that k values of the catalysts further increased with increasing introduction of Ni from NiTiO_3_(0.5)/TiO_2_(TSS) to NiTiO_3_(1.0)/TiO_2_(TSS). NiTiO_3_(1.0)/TiO_2_ (TSS) exhibited the highest visible-light photocatalytic efficiency, which was 2.8 and 8.0 times that of TiO_2_(TSS) and TiO_2_ prepared without templates, respectively. After that, further increase in Ni contents led to a progressive decline of the photocatalytic activity. Especially for NiTiO_3_(3.0)/TiO_2_(TSS), k value was only slightly higher than that of TiO_2_ (TSS), indicating that this improvement of NiTiO_3_ for photocatalytic activity is within an appropriate range. Excessive NiTiO_3_ nanoparticles may cover the surface of TiO_2_ and act as the charge recombination centre of photogenerated carriers, leading to a decrease in active species and thereby reducing photocatalytic activity.

Furthermore, as shown in Figure 3b, the prepared NiTiO_3_/TiO_2_(TSS) photocatalysts exhibited good activity under NIR light (λ = 840~850 nm) without upconversion materials. When NIR light was on, NiTiO_3_ content had a significant impact on the photocatalytic activities of the prepared catalysts. The efficiencies of TC removal over TiO_2_, TiO_2_(TSS), NiTiO_3_(0.5)/TiO_2_(TSS), NiTiO_3_(1.0)/TiO_2_(TSS), NiTiO_3_(1.5)/TiO_2_(TSS), NiTiO_3_(2.0)/TiO_2_ (TSS), and NiTiO_3_(3.0)/TiO_2_(TSS) were 16.5, 33.7, 42.9, 49.7, 55.5, 46.7, and 51.9%, respectively. As shown in Figure S9b, NiTiO_3_(1.5)/TiO_2_(TSS) exhibited higher photocatalytic activities than NiTiO_3_ and Ni/TiO_2_. In order to further evaluate the NIR-light photocatalytic activities of all prepared catalysts, k value for photocatalytic degradation of TC was calculated and plotted in Figure S8b. As shown in Figure 3d, the k value of NiTiO_3_(1.5)/TiO_2_(TSS) is 2.8 and 2.2 times that of TiO_2_ and TiO_2_(TSS), respectively.

Moreover, as shown in Figure S9, NiTiO_3_(1.0)/TiO_2_(TSS) and NiTiO_3_(1.5)/TiO_2_ (TSS) exhibited much higher photocatalytic activity than Ni/TiO_2_ (same Ni amount to NiTiO_3_ (1.0)/TiO_2_(TSS)) and pure NiTiO_3_ under both visible and NIR light irradiation. Table S3 summarizes the reaction conditions and removal efficiency of TiO_2_-based catalysts for photocatalytic degradation of TC that have been reported so far. From the results, NiTiO_3_ (1.0)/TiO_2_(TSS) synthesized in this work exhibits more excellent activity in photocatalytic degradation of TC, which is of great significance for practical applications of TC removal.

**Figure 4:**
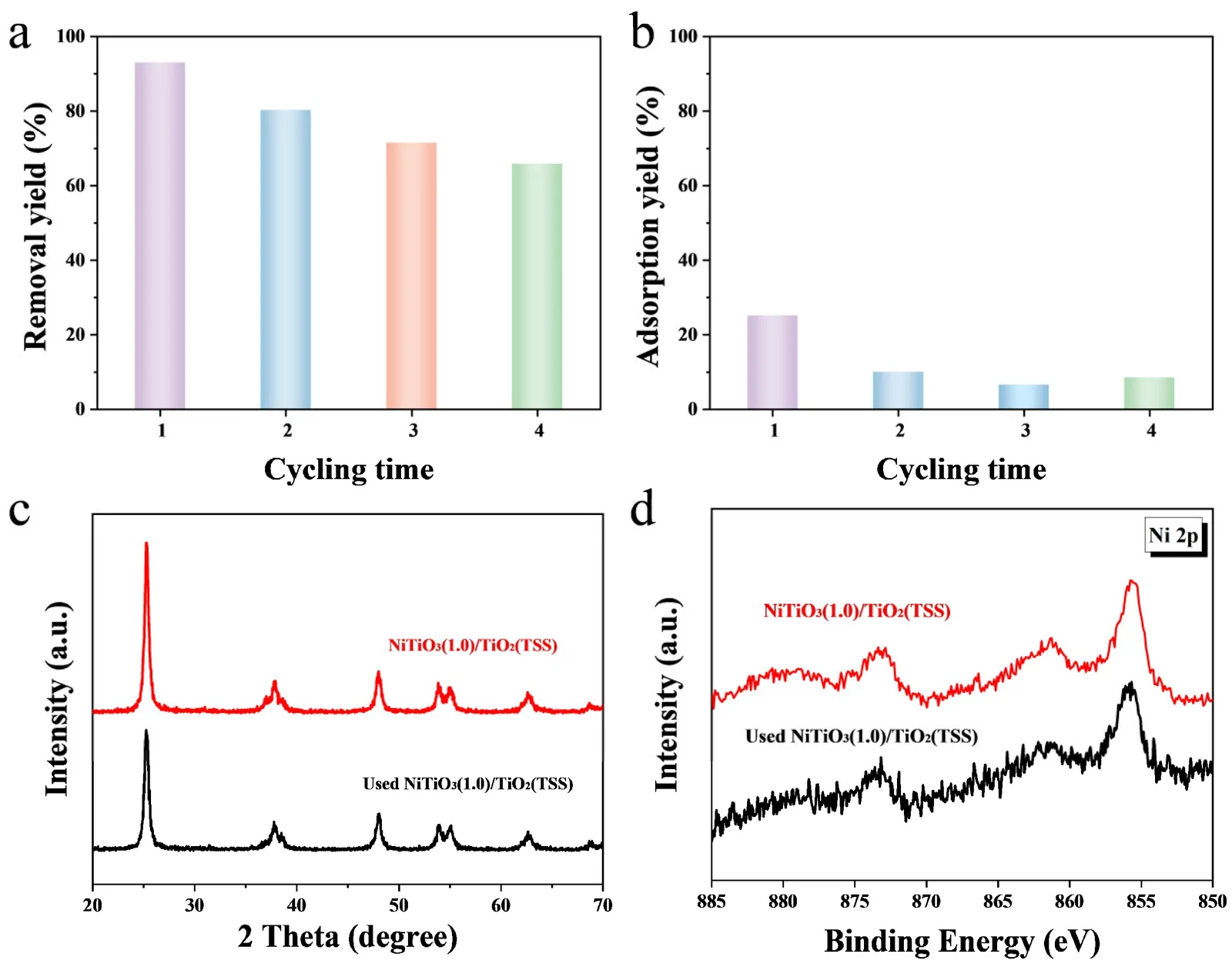
Cycling test data for the removal yield of TC (**a**) and adsorption yield of TC (**b**) over NiTiO_3_(1.0)/TiO_2_(TSS). XRD patterns and (**c**) XPS spectra of Ni 2p of (**d**) the fresh and used NiTiO_3_(1.0)/TiO_2_(TSS).

To evaluate the stability and recyclability of the prepared catalysts, cycling photocatalytic experiments were conducted over NiTiO_3_(1.0)/TiO_2_(TSS) for four cycles under visible light irradiation. As shown in Figure 4a,b, the removal yield of TC decreased from 93% to 69% and the adsorption yield also showed a significant decline from 24% to below 10% after four cycles. The results indicated that the pores of the catalyst were blocked, and the adsorption position was occupied by intermediate products during the degradation process after the first cycle, leading to the decrease in removal yield and adsorption yield. However, after the calculation, it was found that the total amount of TC removed by photocatalytic degradation in the four cycles was relatively close, at 9.9, 10.3, 9.3, and 8.6 mg/g, respectively (in Figure S10). Therefore, NiTiO_3_(1.0)/TiO_2_(TSS) has good stability for photocatalysis. Moreover, the XRD and XPS analysis were carried out to reveal the stability of NiTiO_3_(1.0)/TiO_2_(TSS). As shown in Figure 4c,d, the used NiTiO_3_(1.0)/TiO_2_(TSS) is still single crystalline of anatase and the valence state of Ni does not change, which is the same to the fresh NiTiO_3_(1.0)/TiO_2_(TSS). Thus, the NiTiO_3_ (1.0)/TiO_2_(TSS), with high activity and long-term stability, is potential for degradation of organic pollutants over photocatalytic process in practical applications.

### 3.3. Plausible Mechanism of Photocatalytic Oxidation of Tetracycline

**Figure 5:**
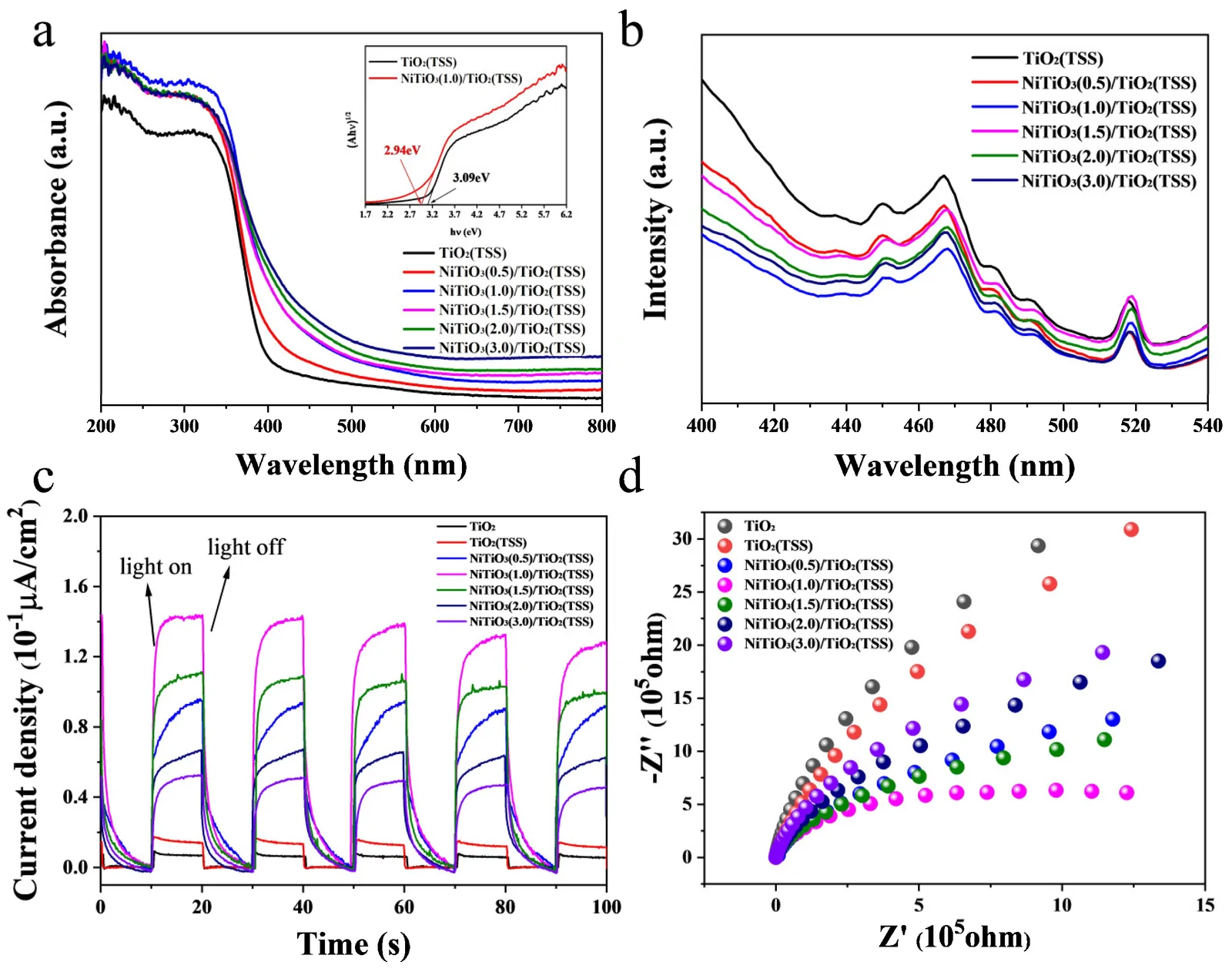
The UV-Vis diffuse reflectance spectra (**a**), PL spectra (**b**) of TiO_2_(TSS) and NiTiO_3_/TiO_2_(TSS). Transient photocurrent response (**c**) and EIS Nyquist plots (**d**) of TiO_2_, TiO_2_(TSS) and NiTiO_3_/TiO_2_ (TSS).

It is common knowledge that light absorption by the material as well as migration of the light-induced electrons and holes are the key factors to control a photocatalytic reaction, which is relevant to the electronic structure characteristics of the material. The UV-Vis diffuse reflectance spectra (DRS) were employed to examine the influence of introducing NiTiO_3_ on optical properties of the prepared photocatalysts. As shown in Figure S11, bare NiTiO_3_ exhibits a distinctive absorption band, signifying elevated absorption coefficients in both the ultraviolet region (250−350 nm) and the infrared region (750−850 nm). Within the visible-light spectrum, a broad absorption edge at 410 nm is observed, attributed to the interaction involving O^2−^/Ti^4+^ charge transfer in NiTiO_3_. Additionally, two subtle absorption peaks at 450 and 510 nm can be attributed to the crystal field splitting phenomenon within NiTiO_3_. It can be observed in Figure 5a that TiO_2_(TSS) exhibits a strong absorption in the ultraviolet region (250−350 nm) [19]. The NiTiO_3_/TiO_2_(TSS) photocatalysts show an absorption peak spectrum that is basically same as that of TiO_2_(TSS), indicating that the load of NiTiO_3_ only has a little effect on the light absorption of TiO_2_(TSS), which may be related to the lower NiTiO_3_ content. With the increase of NiTiO_3_ loading, the light absorption of the composite NiTiO_3_/TiO_2_(TSS) continue to increase and the absorption edge show a red shift to higher wavelength, which is caused by the interaction between NiTiO_3_ and TiO_2_ [48]. The light absorption of synthesized photocatalysts under visible light gradually enhances with the increasing NiTiO_3_/TiO_2_ heterojunction. Therefore, the interaction between NiTiO_3_ and TiO_2_ efficiently promotes the light absorption, which is beneficial to the improvement of photocatalytic activity [51,52]. Notably, NiTiO_3_ (1.0)/TiO_2_(TSS) and NiTiO_3_(1.5)/TiO_2_(TSS) shows stronger absorption and higher red shift than Ni/TiO_2_ in both the ultraviolet region and the infrared region (in Figure S11), accounting for the lower activity of Ni/TiO_2_. Furthermore, the band gap energies of TiO_2_(TSS) and NiTiO_3_/TiO_2_(TSS) are determined based on the Tauc plots derived from spectra of DRS in Figure 5a, indicating that the band gap (Eg) of TiO_2_(TSS) will be influenced by adding NiTiO_3_. The band gap energy of TiO_2_(TSS) is determined to be approximately 3.09 eV, a value in close agreement with the experimental data reported in the literature [53]. With the increase of NiTiO_3_ content, as illustrated in Table S2, the band gap energy gradually decreases and the minimum Eg is 2.78 eV for NiTiO_3_(3.0)/TiO_2_(TSS).

The PL was employed to characterize TiO_2_(TSS) and NiTiO_3_/TiO_2_(TSS). Information such as the efficiency of charge carrier trapping, immigration, and transfer can be obtained by PL. Thus, the PL spectra are often utilized to investigate surface processes involving the electron-hole fate of the semiconductor [54]. The PL intensity is proportional to the recombination rate of the photogenerated electron-hole pairs. As shown in Figure 5b, PL spectra of TiO_2_(TSS) and NiTiO_3_/TiO_2_(TSS) exhibits three main emission peaks at about 450, 467, and 518 nm with excitation at 300 nm, indicating a similarity in crystallite structure between NiTiO_3_/TiO_2_(TSS) and TiO_2_(TSS). In comparison with TiO_2_(TSS), combining with NiTiO_3_ does not affect the spectral position of the peaks, but reduces the relative intensity of the PL spectra. The reducing PL intensity suggests that the interaction between NiTiO_3_ and TiO_2_ contributes to the effective charge separation and efficient inhibition of charge recombination, thus creating a significantly improved photocatalytic activity for the NiTiO_3_/TiO_2_(TSS) composites under visible-light irradiation. Notably, NiTiO_3_(1.0)/TiO_2_(TSS) shows the lowest PL intensity, which is well consistent with its photocatalytic activity.

In order to further understand the generation and transmission process of photogenerated electrons in the synthesized photocatalysts, transient J-t photo response of TiO_2_ prepared without templates, TiO_2_(TSS) and NiTiO_3_/TiO_2_(TSS) were tested under visible-light irradiation. As shown in Figure 5c, the transient photocurrent intensity of NiTiO_3_/TiO_2_(TSS) increases almost exponentially compared to TiO_2_ prepared without templates and TiO_2_(TSS), indicating a higher photocurrent density. It can be attributed to the direct Z-scheme photocatalytic system of NiTiO_3_/TiO_2_(TSS) to absorb photons in the visible-light region and promote the separation and migration of photogenerated electron and hole pairs [48], which is beneficial for photocatalysis and consistent with the photocatalytic performance results. In addition, EIS Nyquist plots are considered as suitable indicators of the charge carrier migration and interfacial transfer/recombination rates [55]. EIS Nyquist plots under visible-light irradiation are shown in Figure 5d. The arc radius of NiTiO_3_(1.0)/TiO_2_(TSS) is the lowest among the prepared catalysts, indicating more effective separation of photogenerated carriers and higher charge mobility across the interface between NiTiO_3_ and TiO_2_, which is consistent with the transient photocurrent response results. Additionally, the transient photocurrent intensity of TiO_2_(TSS) is higher and arc radius is smaller than TiO_2_ prepared without templates, indicating that the introduction of special flaky structure of tobacco stem-silks enhances light-harvesting ability and promotes the separation and migration of photogenerated electron and hole pairs.

**Figure 6:**
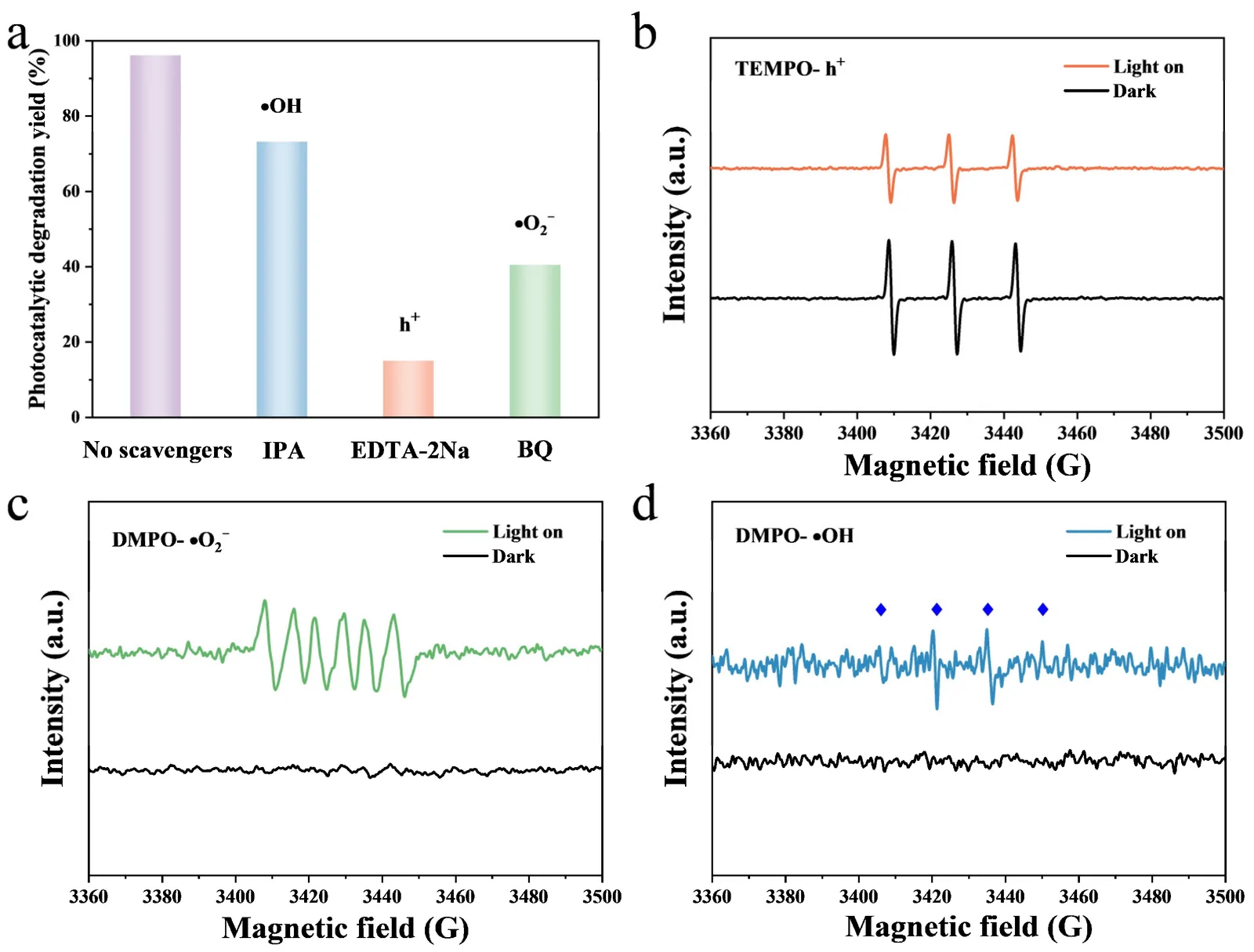
Active oxidative species trapping experiments for degradation of TC (**a**), EPR spectra by adding TEMPO to capture h^+^ (**b**), EPR spectra by adding DMPO to capture •O_2_^−^ and (**c**) EPR spectra by adding DMPO to capture •OH (**d**) under visible light irradiation over NiTiO_3_(1.0)/TiO_2_(TSS).

Radicals trapping experiments were conducted over NiTiO_3_(1.0)/TiO_2_(TSS) to further investigate the possible photocatalytic mechanism. Different kinds of the radical trapping agents were introduced into the photoreactor, which sequestered active species during the photodegradation process and then deactivated their effects. In this way, isopropyl alcohol (IPA), ethylenediaminetetraacetic acid disodium salt (EDTA-2Na), and p-benzoquinone (BQ) were employed as trapping agents for hydroxy radicals (•OH), photogenerated(h+), and superoxide radicals (•O_2_^−^), respectively. The results of the main reactive oxidation species (ROS) responsible for TC photodegradation process are shown in Figure 6a. The photodegradation efficiency exhibited slight decrease in the presence of IPA (73.2%) and was hampered in the presence of EDTA-2Na (15.9%) and BQ (47.1%) with a significant drop, implying that h^+^, •OH and •O_2_^−^ contribute to TC photodegradation. Moreover, h^+^ and •O_2_^−^ are dominant ROS in the photodegradation process. In order to further understand the ROS during the visible-light photocatalytic degradation of TC by NiTiO_3_(1.0)/TiO_2_(TSS), the presence of •OH, h^+^, and •O_2_^−^ was identified by EPR. 2,2,6,6-Tetramethylpiperidoxyl (TEMPO) was used to capture h^+^, as shown in Figure 6b. A clear TEMPO signal was detected under dark conditions. After 6 min of visible light irradiation, the TEMPO signal intensity significantly decreased, which was due to the consumption of TEMPO by h^+^. The results indicated that NiTiO_3_(1.0)/TiO_2_(TSS) generated photogenerated holes on the surface under visible light irradiation [56,57]. As shown in Figure 6c,d, the presence of •O_2_^−^ and •OH was verified by 5,5-Dimethyl-1-Pyrroline-N-oxide (DMPO). No signal was detected under dark condition. The characteristic signal of DMPO-•O_2_^−^ adduct was observed after 6 min of visible light irradiation, which supported the generation of •O_2_^−^ [58,59]. Similarly, four weaker signal peaks generated by DMPO-•OH were detected after 6 min of visible light irradiation, which supported the generation of •OH [59,60]. The EPR results indicated that h+, and •O_2_^−^ played a dominant role in the degradation of TC, which was consistent with the results of scavenger experiment.

**Figure 7:**
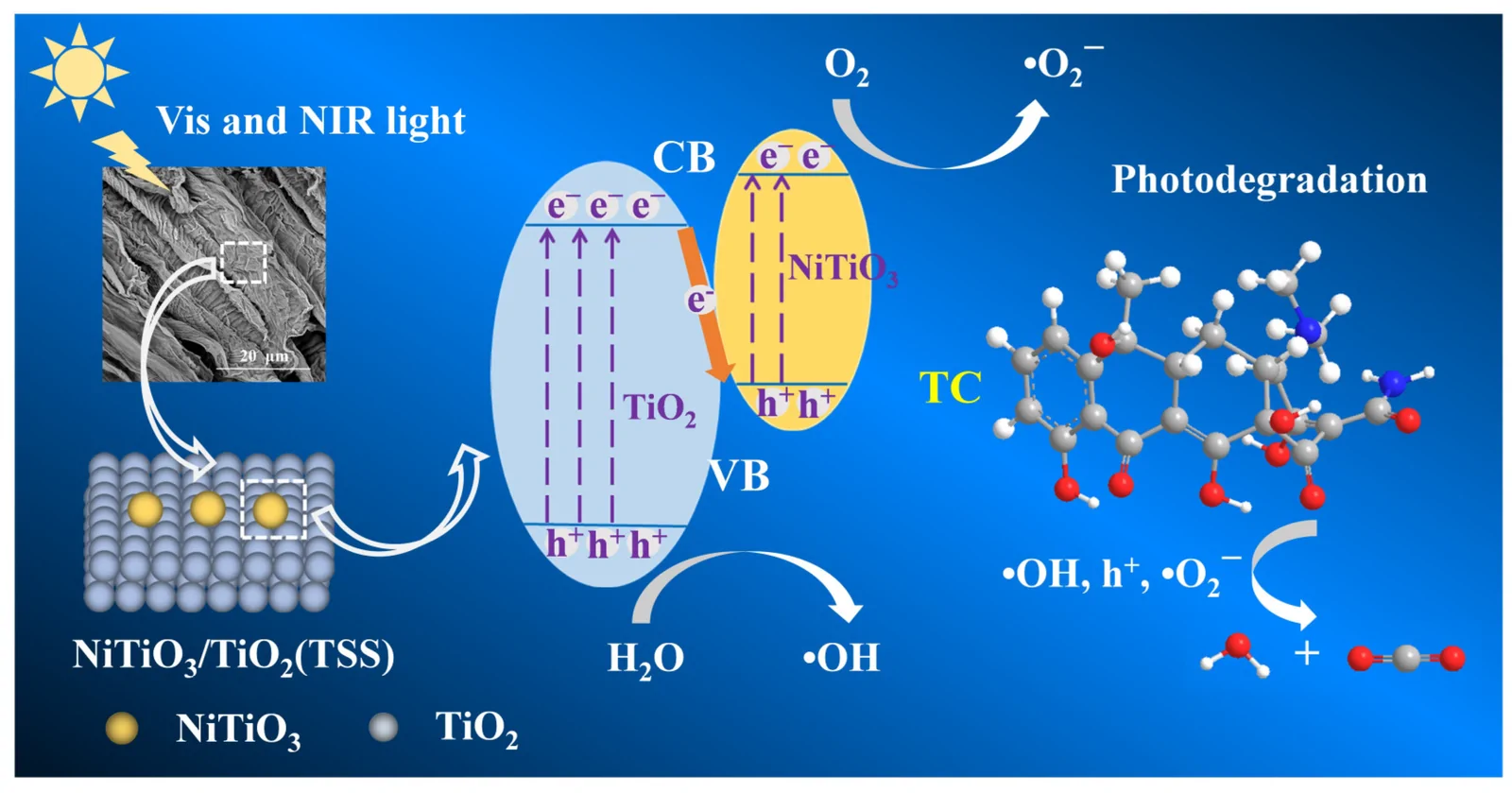
Reaction mechanism of visible light-driven photocatalytic degradation of TC of NiTiO_3_(1.0)/TiO_2_(TSS).

For NiTiO_3_(1.0)/TiO_2_(TSS), Figure S12 shows that the maximum value of valence band (VB) is 2.84 eV, slightly less than the VB edge potential of general anatase TiO_2_ in reported literature [53]. Combined with the corresponding Eg (3.09 eV), the minimum value of conduction band (CB) can be calculated using the formula ECB = EVB − Eg, which is equal to −0.25 eV. Based on the forementioned results, Figure 7 displays a scheme of the electronic band structures over NiTiO_3_(1.0)/TiO_2_(TSS). The relative band structure between NiTiO_3_ and TiO_2_ allows the formation of a direct Z-scheme heterojunction with strong interactions [48], which can enhance the visible and NIR light absorption and promote efficient charge separation and transfer. In such NiTiO_3_-TiO_2_ system, once irradiated by visible light, photogenerated electrons in the CB of TiO_2_ recombine with photogenerated holes in the VB of NiTiO_3_, resulting the efficient charge separation and migration of the direct Z-scheme heterojunction. This is conducive to the accumulation of abundant free electrons in the CB of NiTiO_3_, robustly inducing the generation of the active species of •O_2_^−^. Additionally, part of photogenerated holes with strong oxidation abilities accumulated in the VB of TiO_2_ can react with H_2_O molecules to generate the •OH. The photogenerated ROS that participate in this system are responsible for the highly effective TC degradation. Therefore, the introduction of special flaky structure of tobacco stem-silks and the formation of direct Z-scheme NiTiO_3_/TiO_2_ heterojunction can promote photogenerated carriers with strong oxide-redox abilities to drive photocatalytic reaction [51]. NiTiO_3_/TiO_2_(TSS) composites at optimal NiTiO_3_ loading condition show a significantly increased photocatalytic activity. Similar Z-scheme photocatalytic systems have also been reported, such as TiO_2_/Rh [61], TiO_2_/CdS [62], and CdS/cobalt-benzimidazole [63].

### 3.4. Transformation Products of Tetracycline

**Figure 8:**
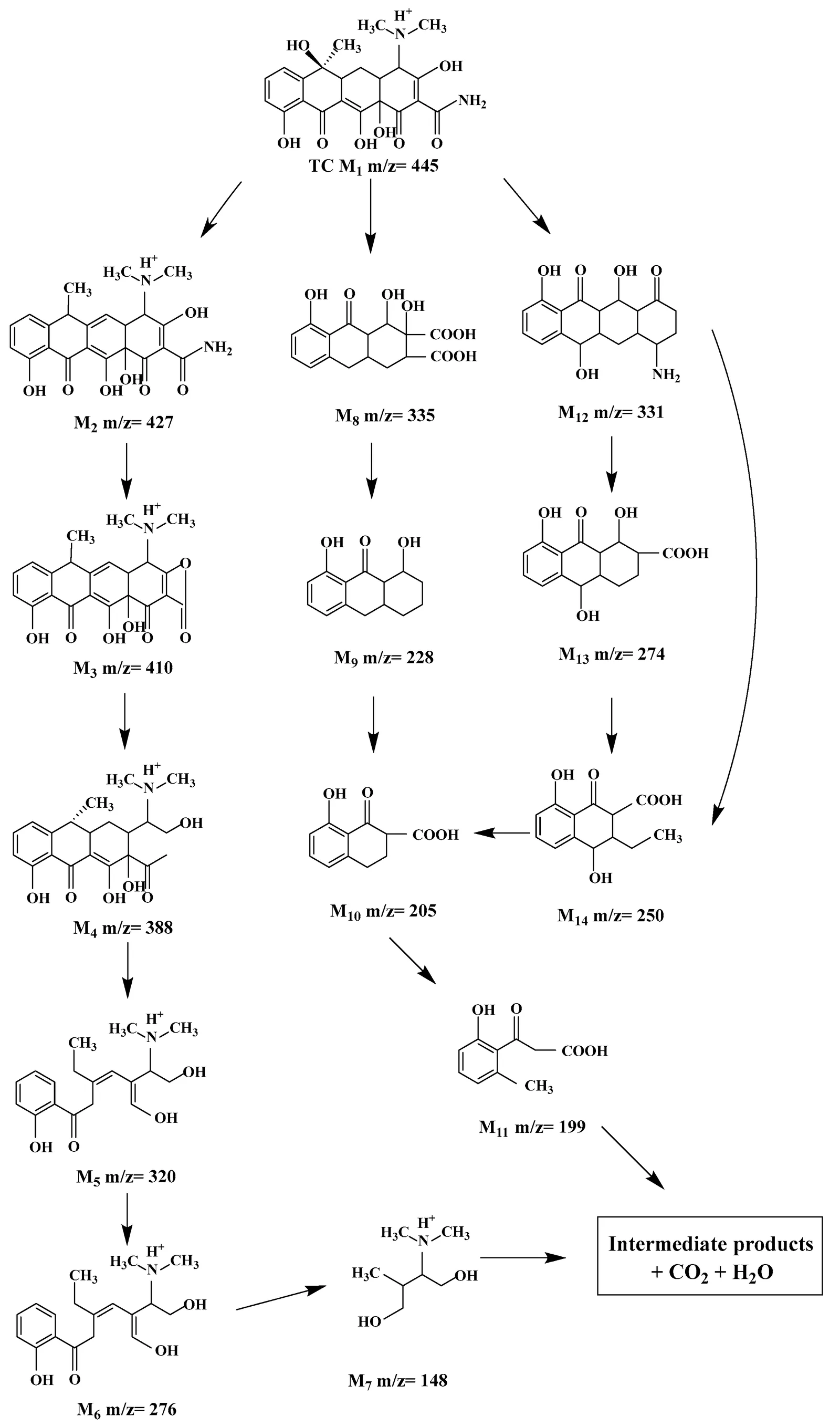
The proposed degradation pathway of TC over NiTiO_3_(1.0)/TiO_2_(TSS).

The UPLC-MS/MS transformation was performed to investigate the reaction pathways of TC degradation over NiTiO_3_(1.0)/TiO_2_(TSS). In Figure S13, the mass spectra of TC and possible intermediates at different retention times can be seen. The mass peak at *m*/*z* 445 corresponds to TC, and its intensity decreased gradually with the process of TC degradation, indicating that TC was attacked and destroyed by ROS. TC molecules were adsorbed on the surface of composites and then attacked by active species (h^+^, •OH, and •O_2_**^−^**), which led to the formation of varieties of intermediates. As shown in Figure 8, there were three main pathways in TC degradation based on UPLC–MS/MS results. The first pathway showed that the intermediate product of M2 (*m*/*z* = 427) was generated from the dehydroxylation of TC. Under the attack of continuous active species, M2 was decomposed into low-molecular-weight organic compounds (M3–M7) via deamination and ring opening reaction. This process was mainly initiated by •OH and •O_2_^−^ [64]. The second and third pathways showed that the compounds of M8 (*m*/*z* = 335) and M12 *m*/*z* = 331) originated from deamination, dehydration, and demethylation of TC. As the reaction proceeded, intermediate products M9–M10 and M13–M14 were generated due to decarboxylation, hydroxyl oxidation, and C-C bond breaking. With the deepening of oxidation, the intermediate M11 was formed through C-C bond fracture [65]. Finally, some intermediates were completely oxidized into CO_2_ and H_2_O molecules.

## 4. Discussion

A novel NiTiO_3_/TiO_2_(TSS) vis-NIR photocatalysts was prepared by using waste tobacco stem-silks as biotemplates via a facile one-pot method. NiTiO_3_(1.0)/TiO_2_(TSS) with mass percent of Ni 1.0% exhibited very high visible-light photocatalytic efficiency in photodegradation of tetracycline hydrochloride, which is 8.0 and 2.3 times higher than TiO_2_ prepared without templates and TiO_2_(TSS) templated by tobacco stem-silks without impregnation of Ni, respectively. In addition, NiTiO_3_(1.0)/TiO_2_(TSS) synthesized in this work exhibited more excellent activity in photocatalytic degradation of TC compared to the reported TiO_2_-based catalysts, which is of great significance and potential for practical applications of TC removal. More importantly, NiTiO_3_(1.5)/TiO_2_(TSS) even exhibited good activity under NIR light (λ = 840~850 nm) without upconversion materials or plasmon-assisted noble metals, which is 2.8 and 2.2 times than TiO_2_ and TiO_2_(TSS), respectively. The direct Z-scheme NiTiO_3_/TiO_2_ heterojunctions with strong interactions and the special flaky structure of tobacco stem-silks can enhance light-harvesting ability and promote efficient charge separation and transfer, which boosts the photocatalytic activities of NiTiO_3_/TiO_2_(TSS) under visible and even NIR light irradiation. Furthermore, the possible three degradation pathways of TC and photocatalytic mechanism of NiTiO_3_/TiO_2_(TSS) were proposed by radicals trapping experiments and UPLC-MS/MS results. The generated h^+^, and •O_2_**^−^** play a dominant role in photocatalytic degradation of TC. This work provides a new idea for the multifunctional utilization of waste tobacco stem-silks and the synthesis of novel vis-NIR photocatalysts for potential application in wastewater treatment.

## Data Availability

The original contributions presented in this study are included in the article/supplementary material. Further inquiries can be directed to the corresponding author.

## Supplementary Materials

The following supporting information can be downloaded at: https://www.mdpi.com/article/10.3390/nano15020080/s1, Figure S1: Standard curve for TC quantification by HPLC; Figure S2: XRD patterns (a) and XPS spectra of Ni 2p (b) of NiTiO_3_(3.0)/TiO_2_(TSS) and NiTiO_3_(5.0)/TiO_2_(TSS); Figure S3: XPS spectra of full spectra (a) and C 1s (b) of NiTiO_3_/TiO_2_(TSS); Figure S4: SEM images of TiO_2_ prepared without templates (a), TiO_2_(TSS) (b), the original tobacco stem-silks (c) and NiTiO_3_(1.0)/TiO_2_(TSS) (d); Figure S5: EDX image of NiTiO_3_(1.0)/TiO_2_(TSS); Figure S6: TEM images of TiO_2_ prepared without templates (a) and TiO_2_(TSS) (c), and HR-TEM images of TiO_2_ prepared without templates (c) and TiO_2_(TSS) (d); Figure S7: N_2_ adsorption–desorption isotherms for (TSS) (a), NiTiO_3_ (0.5)/TiO_2_(TSS) (b), NiTiO_3_(1.0)/TiO_2_ (TSS) (c), NiTiO_3_(1.5)/TiO_2_(TSS) (d), NiTiO_3_(2.0)/TiO_2_(TSS) (e) and NiTiO_3_(3.0)/TiO_2_(TSS) (f); Figure S8: Plot of ln(Ce/C) versus the irradiation time with all prepared photocatalysts under visible light irradiation (a) and NIR light irradiation (b); Figure S9: Removal curves of TC over the photocatalysts for comparation under visible-light irradiation (a) and under NIR (840–850 nm) light irradiation (b); Figure S10: The calculated total amount of TC removed by photocatalytic degradation in the four cycles over NiTiO_3_(1.0)/TiO_2_(TSS); Figure S11: The UV-Vis-NIR diffuse reflectance spectra of TiO_2_(TSS), Ni/TiO_2_, NiTiO_3_(1.0)/TiO_2_(TSS), NiTiO_3_(1.5)/TiO_2_(TSS) and NiTiO_3_; Figure S12: XPS valence band spectra and energy band diagrams (inset) of NiTiO_3_(1.0)/TiO_2_ (TSS); Figure S13: UPLC-MS/MS spectra of degradation products under visible light over NiTiO_3_(1.0)/TiO_2_(TSS) at the reaction time of 10 min. Table S1: HPLC conditions for TC quantification; Table S2: Physicochemical property of as-prepared samples; Table S3: Comparison of photocatalytic activity for removal of TC over TiO_2_-based photocatalysts.
