# Supplementary material for: One-Pot Synthesis and Enhanced Vis-NIR Photocatalytic Activity of NiTiO3/TiO2 Templated by Waste Tobacco Stem-Silks
Source: Nanomaterials (Basel). 2025 Jan 7;15(2):80. doi: 10.3390/nano15020080 (PMC11767961; doi:10.3390/nano15020080)
Supplement: Supplementary file 1 [file nanomaterials-15-00080-s001.zip › nanomaterials-3373269-supplementary.pdf]

Supplementary Information

One-pot synthesis and enhanced Vis-NIR photocatalytic activity of $NiTiO_3/TiO_2$ templated by waste tobacco stem-silks

Weidong Yuan[1], Xiaohong Chen[1], Yi Zhao, Ying Fang, Han Zhang,

Daomei Chen, Jiaqiang Wang*

* Corresponding author. Tel.: +86-871-65031567

Fax: +86-871-65031567

E-mail address: jqwang@ynu.edu.cn (J. Wang)

Table of Contents

Fig S1. Standard curve for TC quantification by HPLC.

Fig S2. XPS spectra of full spectra (a) and C 1s (b) of $NiTiO_3/TiO_2$(TSS).

Fig S3. SEM images of TiO_2 prepared without templates (a), TiO_2(TSS) (b), the original tobacco stem-silks (c) and $NiTiO_3$(1.0)/TiO_2(TSS) (d).

Fig S4. EDX image of $NiTiO_3$(1.0)/TiO_2(TSS).

Fig S5. TEM images of TiO_2 prepared without templates (a) and TiO_2(TSS) (c), and HR-TEM images of TiO_2 prepared without templates (c) and TiO_2(TSS) (d).

Fig S6. N_2 adsorption-desorption isotherms for TiO_2(TSS) (a), $NiTiO_3$(0.5)/TiO_2(TSS) (b), $NiTiO_3$(1.0)/TiO_2(TSS) (c), $NiTiO_3$(1.5)/TiO_2(TSS) (d), $NiTiO_3$(2.0)/TiO_2(TSS) (e) and $NiTiO_3$(3.0)/TiO_2(TSS) (f).

Fig S7. Plot of $\ln(C_e/C)$ versus the irradiation time with the prepared photocatalysts

under visible-light irradiation (a) and NIR (840-850 nm) light irradiation (b).

Fig S8. Removal curves of TC over the photocatalysts for comparation under visible-light irradiation (a) and under NIR (840-850 nm) light irradiation (b).

Fig.S9. The calculated total amount of TC removed by photocatalytic degradation in the four cycles over $NiTiO_3$(1.0)/TiO_2(TSS).

Fig S10. The UV-vis-NIR diffuse reflectance spectra of TiO_2(TSS), Ni/TiO_2, $NiTiO_3$(1.0)/TiO_2(TSS), $NiTiO_3$(1.5)/TiO_2(TSS) and $NiTiO_3$.

Fig S11. XPS valence band spectra and energy band diagrams (inset) of $NiTiO_3$(1.0)/TiO_2(TSS).

Fig S12. UPLC–MS/MS spectra of degradation products under visible light over $NiTiO_3$(1.0)/TiO_2(TSS) at the reaction time of 10 min.

Table S1. HPLC conditions for TC quantification.

Table S2. Physicochemical property of as-prepared samples.

Table S3. Comparison of photocatalytic activities for removal of TC over TiO_2-based photocatalysts.

Reference

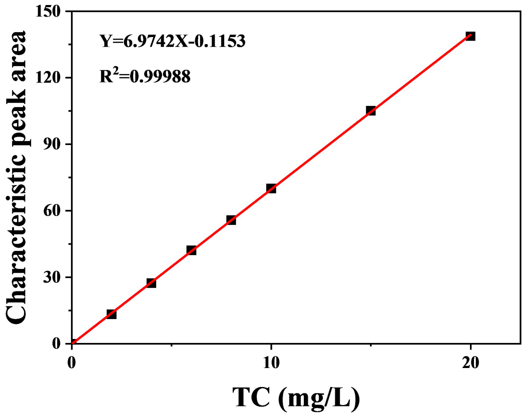

Fig S1. Standard curve for TC quantification by HPLC.

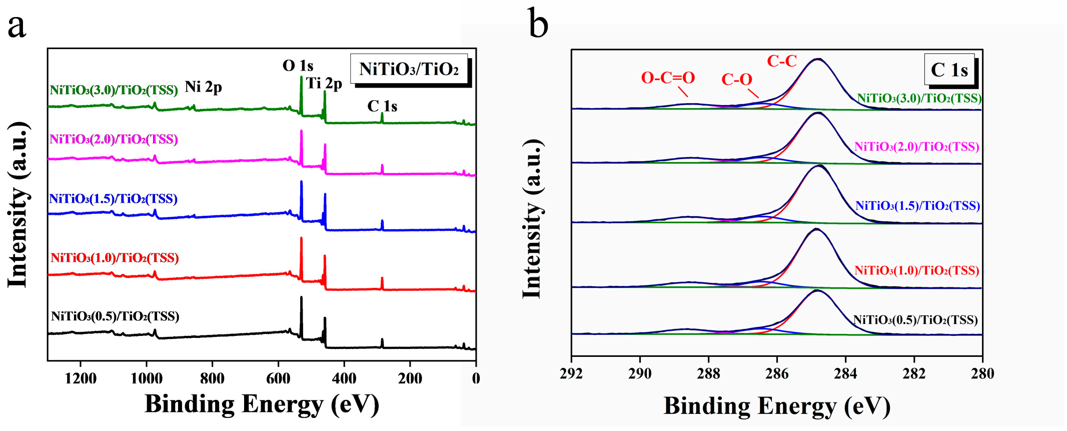

Fig S2. XPS spectra of full spectra (a) and C 1s (b) of $NiTiO_3/TiO_2$(TSS).

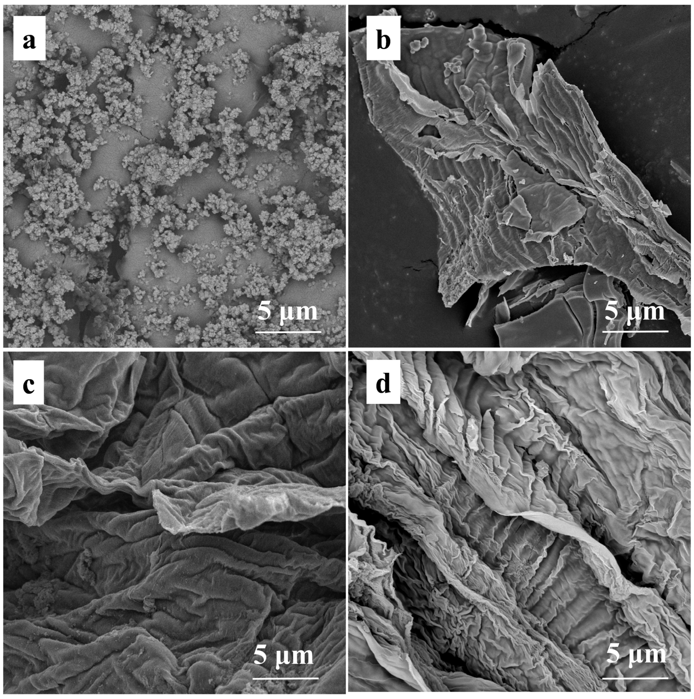

Fig S3. SEM images of TiO_2 prepared without templates **(a)**, TiO_2(TSS) **(b)**, the original tobacco stem-silks **(c)** and $NiTiO_3$(1.0)/TiO_2(TSS) **(d)**.

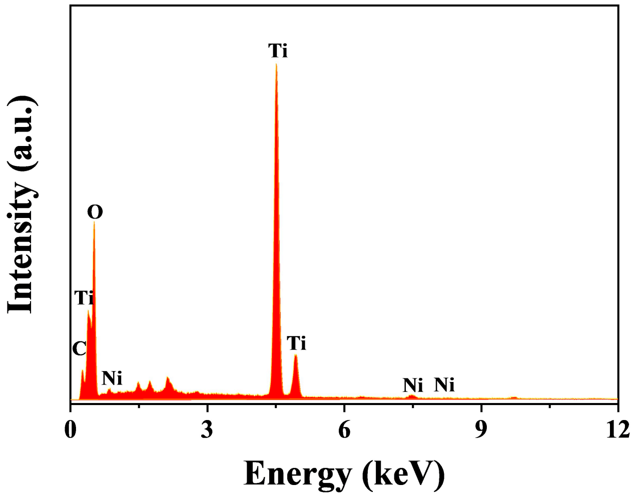

Fig. S4. EDX image of $NiTiO_3$(1.0)/TiO_2(TSS).

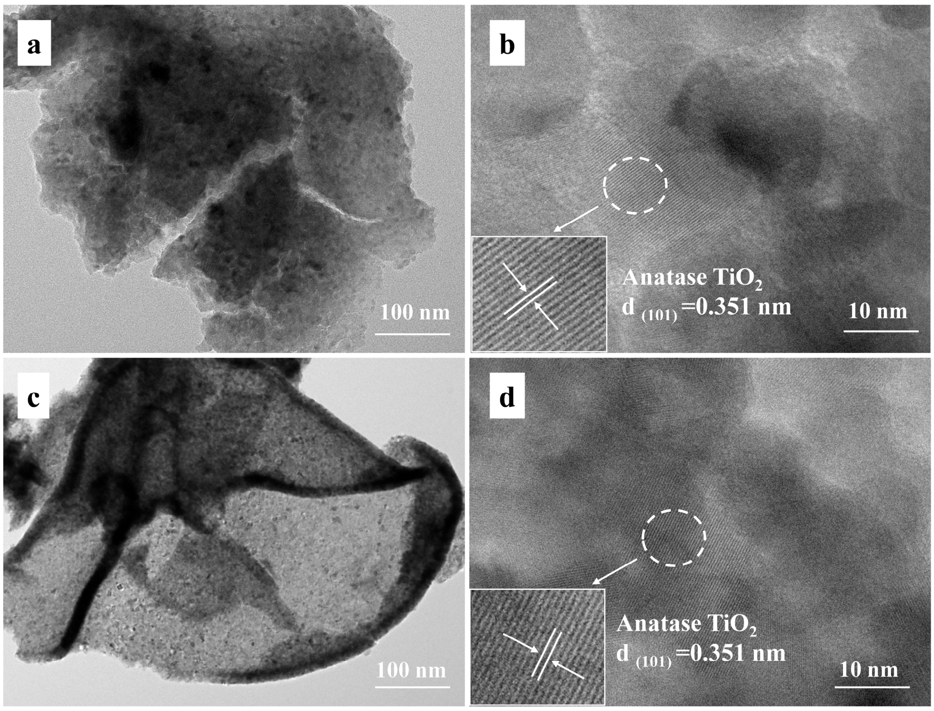

Fig S5. TEM images of TiO_2 prepared without templates **(a)** and TiO_2(TSS) **(c)**, and HR-TEM images of TiO_2 prepared without templates **(b)** and TiO_2(TSS) **(d)**.

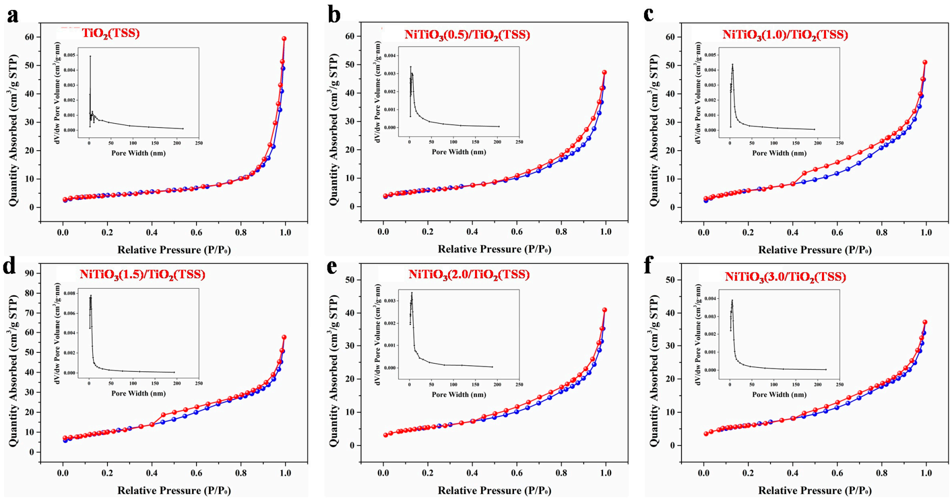

Fig 6. N_2 adsorption-desorption isotherms for TiO_2(TSS) (a), $NiTiO_3$(0.5)/TiO_2(TSS) (b), $NiTiO_3$(1.0)/TiO_2(TSS) (c), $NiTiO_3$(1.5)/TiO_2(TSS) (d), $NiTiO_3$(2.0)/TiO_2(TSS) (e) and $NiTiO_3$(3.0)/TiO_2(TSS) (f).

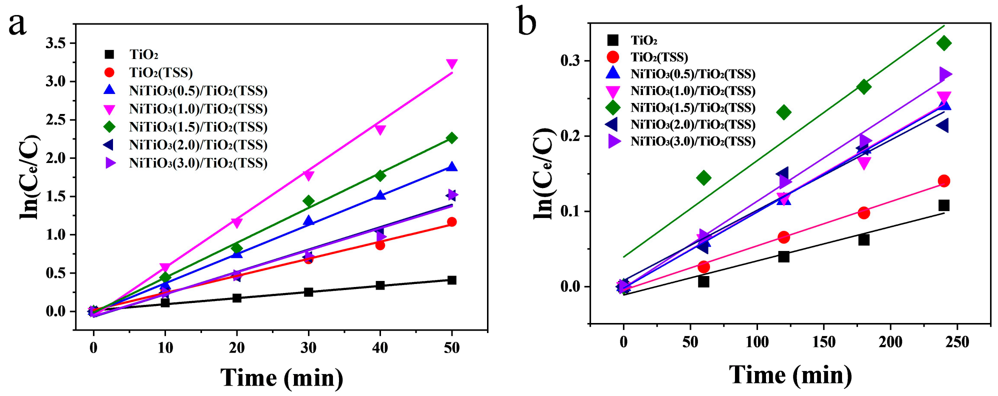

Fig. S7. Plot of $\ln(C_e/C)$ versus the irradiation time with the prepared photocatalysts under visible-light irradiation (a) and NIR (840-850 nm) light irradiation (b).

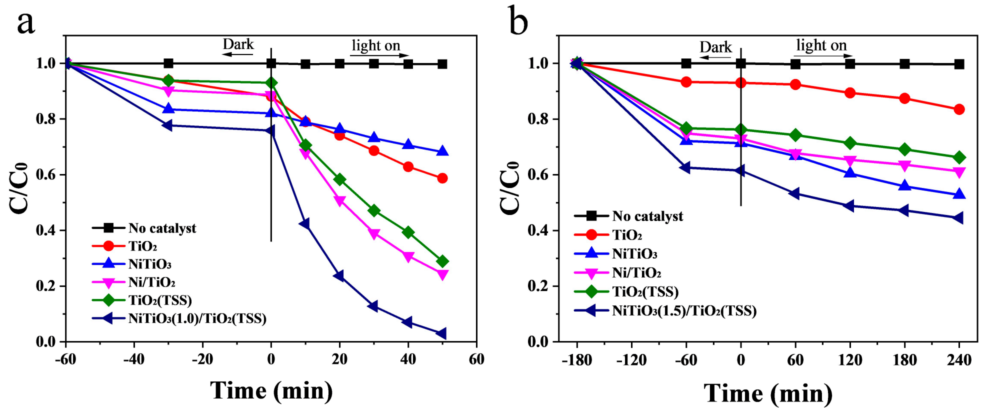

Fig S8. Removal curves of TC over the photocatalysts for comparation under visible-light irradiation (a) and under NIR (840-850 nm) light irradiation (b).

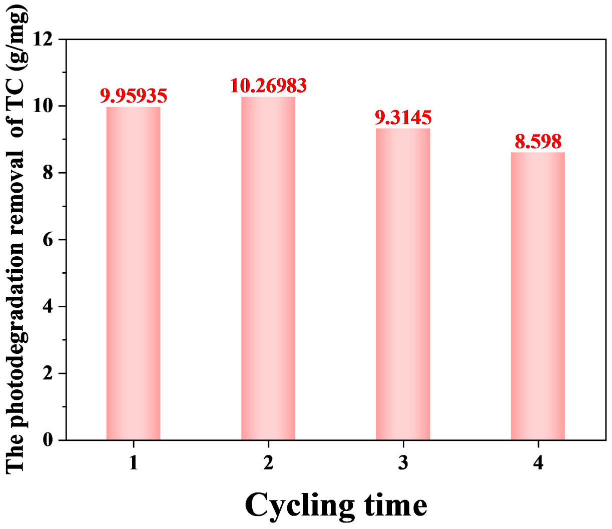

Fig S9. The calculated total amount of TC removed by photocatalytic degradation in the four cycles over $NiTiO_3(1.0)/TiO_2(TSS)$.

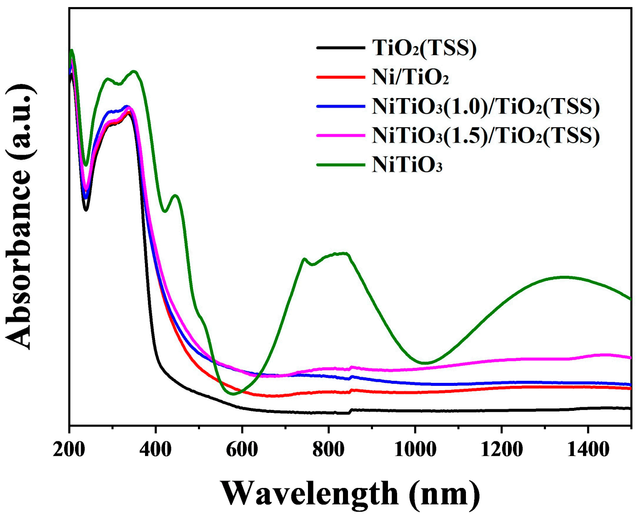

Fig S10. The UV-vis-NIR diffuse reflectance spectra of $TiO_2(TSS)$, Ni/TiO_2, $NiTiO_3(1.0)/TiO_2(TSS)$, $NiTiO_3(1.5)/TiO_2(TSS)$ and $NiTiO_3$.

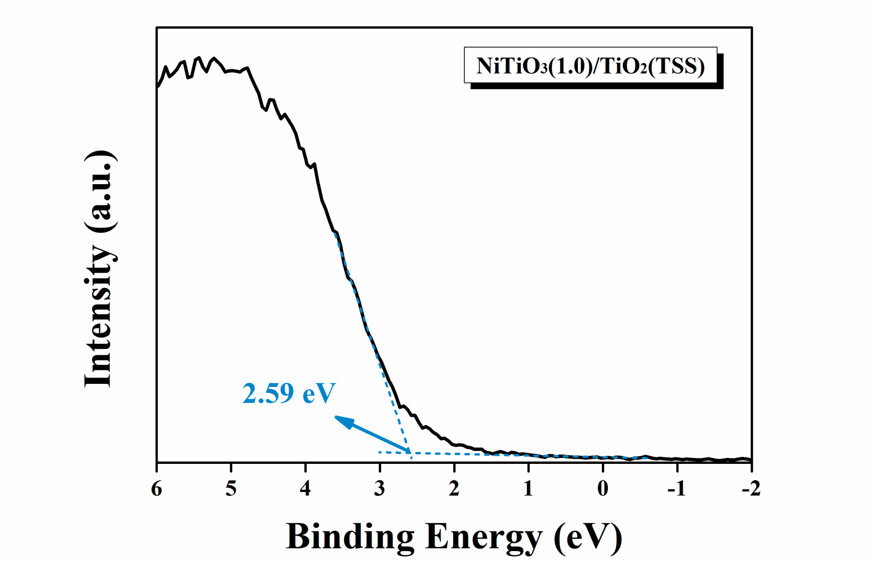

Fig S11. XPS valence band spectra and energy band diagrams (inset) of $NiTiO_3$(1.0)/TiO_2(TSS).

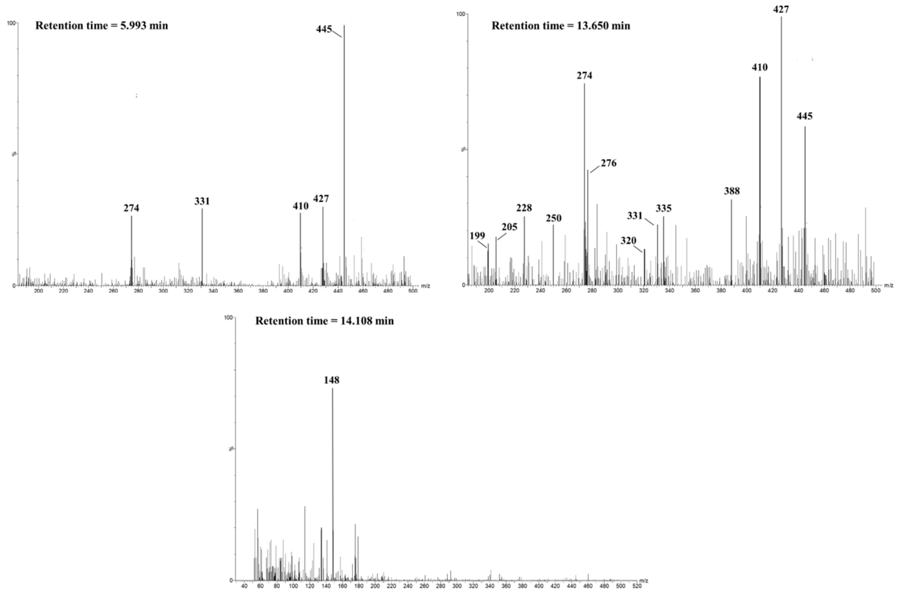

Fig S12. UPLC–MS/MS spectra of degradation products under visible light over $NiTiO_3$(1.0)/TiO_2(TSS) at the reaction time of 10 min.

Table S1. HPLC conditions for TC quantification.

Time (min)	A (%)	B (%)	Flow rate (mL/min)	Maximum pressure limit (bar)
0.0	78.0	22.0	1.0	400.0
3.0	58.0	42.0	1.0	400.0

Table S2. Physicochemical property of as-prepared samples.

Sample	Pore volumn (cm^3/g)	Pore size (nm)	S_{BET} (m^2/g)	Band gap energy (eV)	k (min^{-1})	Ni content (at. %)
TiO_2(TSS)	0.09	11.8	20.9	3.09	0.022	0
$NiTiO_3$(0.5)/TiO_2(TSS)	0.07	27.9	15.4	3.07	0.038	0.52
$NiTiO_3$(1.0)/TiO_2(TSS)	0.09	9.9	23	2.94	0.064	1.22
$NiTiO_3$(1.5)/TiO_2(TSS)	0.10	7.8	37.1	2.92	0.046	1.83
$NiTiO_3$(2.0)/TiO_2(TSS)	0.07	10.3	19.7	2.85	0.029	2.68
$NiTiO_3$(3.0)/TiO_2(TSS)	0.06	8.8	22.2	2.78	0.028	4.48

*a. Ni content was determined by XPS analysis.

Table S3. Comparison of photocatalytic activities for removal of TC over TiO_2-based photocatalysts.

**Sample**	**Light Source and Irradiation Time**	**Catalyst**	**TC**	**Removal Efficiency**	**Ref.**
TiO_{2-x}/ultrathin g-C_3N_4/TiO_{2-x}	300 W Xeon-lamp, visible light, 1 h	1.0 g/L	10 mg/L	87.7%	[1]
N-TiO_2/rGO	300 W Xe-arc lamp, visible light, 1 h	1.0 g/L	10 mg/L	98%	[2]
TiO_2/g-C_3N_4	Xenon lamp full spectrum, 25min	1.0 g/L	20 mg/L	100%	[3]
TiO_2/Fe_2O_3/CNTs	300 W Xeon lamp, visible light, 90 min	1.0 g/L	20 mg/L	89.41%	[4]
CdS-TiO_2	500 W Xenon lamp, visible light, 8 h	1.0 g/L	50 mg/L	87.06%	[5]
$MnCo_2O_{4.5}$/TiO_2	500 W Xenon lamp, visible light, 2h	/	10 mg/L	93.1%	[6]
AgBr-TiO_2-Pal	300 W Xennon lamp, visible light, 2h	0.5 g/L	10 mg/L	90%	[7]
Cu_2O-TiO_2-Pal	500 W Xenon lamp (solar light), 4 h	1.0 g/L	30 mg/L	81.45%	[8]
$NiTiO_3$(1.0)/TiO_2(TSS)	5 W LED lamp, visible light, 50min	0.6 g/L	10 mg/L	97%	This work

References

[1] J. Ni, W. Wang, D. Liu, Q. Zhu, J. Jia, J. Tian, Z. Li, X. Wang, Z. Xing, Oxygen vacancy-mediated sandwich-structural TiO_{2-x}/ultrathin g-C_3N_4/TiO_{2-x} direct Z-scheme heterojunction visible-light-driven photocatalyst for efficient removal of high toxic tetracycline antibiotics, J. Hazard. Mater. 408 (2021) 124432. https://doi.org/10.1016/j.jhazmat.2020.124432.

[2] X. Tang, Z. Wang, Y. Wang, Visible active N-doped TiO_2/reduced graphene oxide for the degradation of tetracycline hydrochloride, Chem. Phys. Lett. 691 (2018) 408–414. https://doi.org/10.1016/j.cplett.2017.11.037.

[3] W. Wang, J. Fang, S. Shao, M. Lai, C. Lu, Compact and uniform TiO_2@g-C_3N_4 core-shell quantum heterojunction for photocatalytic degradation of tetracycline antibiotics, Appl. Catal. B Environ. 217 (2017) 57–64. https://doi.org/10.1016/j.apcatb.2017.05.037.

[4] C. Lu, W. Guan, G. Zhang, L. Ye, Y. Zhou, X. Zhang, TiO_2/Fe_2O_3/CNTs magnetic photocatalyst: A fast and convenient synthesis and visible-light-driven photocatalytic degradation of tetracycline, Micro Nano Lett. 8 (2013) 749–752. https://doi.org/10.1049/mnl.2013.0428.

[5] W. Li, H. Ding, H. Ji, W. Dai, J. Guo, G. Du, Photocatalytic degradation of tetracycline hydrochloride via a CdS-TiO_2 heterostructure composite under visible light irradiation, Nanomaterials. 8 (2018). https://doi.org/10.3390/nano8060415.

[6] Y. Bi, J. Li, C. Dong, W. Mu, X. Han, Rational Construction of $MnCo_2O_{4.5}$ Deposited TiO_2 Nanotube Array Heterostructures with Enhanced Photocatalytic Degradation of Tetracycline, ChemPhotoChem. 4 (2020) 366–372. https://doi.org/10.1002/cptc.201900283.

[7] Y. Shi, Z. Yan, Y. Xu, T. Tian, J. Zhang, J. Pang, X. Peng, Q. Zhang, M. Shao, W. Tan, H. Li, Q. Xiong, Visible-light-driven AgBr–TiO_2-Palygorskite photocatalyst with excellent photocatalytic activity for tetracycline hydrochloride, J. Clean. Prod. 277 (2020) 124021. https://doi.org/10.1016/j.jclepro.2020.124021.

[8] Y. Shi, Z. Yang, B. Wang, H. An, Z. Chen, H. Cui, Adsorption and photocatalytic degradation of tetracycline hydrochloride using a palygorskite-supported Cu_2O-TiO_2 composite, Appl. Clay Sci. 119 (2016) 311–320. https://doi.org/10.1016/j.clay.2015.10.033.
